# Mutations in *Ovis aries TMEM154* are associated with lower small ruminant lentivirus proviral concentration in one sheep flock

**DOI:** 10.1111/age.12181

**Published:** 2014-06-17

**Authors:** F A Alshanbari, M R Mousel, J O Reynolds, L M Herrmann-Hoesing, M A Highland, G S Lewis, S N White

**Affiliations:** * Department of Veterinary Microbiology and Pathology, Washington State UniversityPullman, WA, 99164, USA; † USDA-ARS US Sheep Experiment StationDubois, ID, 83423, USA; ‡ USDA-ARS Animal Disease Research UnitPullman, WA, 99164, USA

**Keywords:** *chemokine (C-C motif) receptor 5*, disease susceptibility, domestic sheep, maedi–visna virus, ovine lentivirus, ovine progressive pneumonia virus, *transmembrane protein 154*, viremia

## Abstract

Small ruminant lentivirus (SRLV), also called ovine progressive pneumonia virus or maedi-visna, is present in 24% of US sheep. Like human immunodeficiency virus, SRLV is a macrophage-tropic lentivirus that causes lifelong infection. The production impacts from SRLV are due to a range of disease symptoms, including pneumonia, arthritis, mastitis, body condition wasting and encephalitis. There is no cure and no effective vaccine for preventing SRLV infection. However, breed differences in prevalence and proviral concentration indicate a genetic basis for susceptibility to SRLV. Animals with high blood proviral concentration show increased tissue lesion severity, so proviral concentration represents a live animal test for control post-infection in terms of proviral replication and disease severity. Recently, it was found that sheep with two copies of *TMEM154* haplotype 1 (encoding lysine at position 35) had lower odds of SRLV infection. In this study, we examined the relationship between SRLV control post-infection and variants in two genes, *TMEM154* and *CCR5*, in four flocks containing 1403 SRLV-positive sheep. We found two copies of *TMEM154* haplotype 1 were associated with lower SRLV proviral concentration in one flock (*P* < 0.02). This identified the same favorable diplotype for SRLV control post-infection as for odds of infection. However, frequencies of haplotypes 2 and 3 were too low in the other three flocks to test. The *CCR5* promoter deletion did not have consistent association with SRLV proviral concentration. Future work in flocks with more balanced allele frequencies is needed to confirm or refute *TMEM154* association with control of SRLV post-infection.

## Introduction

Small ruminant lentivirus (SRLV), also known as maedi–visna virus or ovine progressive pneumonia virus, is a common pathogen in the US, where an estimated 24% of sheep are seropositive (Cutlip *et al*. 1992). Transmission of SRLV is thought to occur via the respiratory route with one major source being colostrum/milk from infected ewes (Reina *et al*. 2009), and the virus can be transmitted throughout an animal's lifespan (De Boer *et al*. 1979). In addition to inducing lifelong infection, SRLV causes varying clinical manifestations of pneumonia, arthritis, mastitis, cachexia, dyspnea and/or encephalitis (Blacklaws 2012). Small ruminant lentivirus disease symptoms generally become more evident with advancing age, and infected ewes are culled approximately one year earlier than uninfected ewes (Peterhans *et al*. 2004), which can be a large proportion of a ewe's productive lifetime (Annett *et al*. 2011; Byun *et al*. 2012). Additional adverse sheep production impacts from SRLV include reduced birth rates, birth weights and lamb growth as well as import restrictions (Keen *et al*. 1997; Arsenault *et al*. 2003; Reina *et al*. 2009). Methods of controlling or preventing SRLV infection, such as (i) repeated serological testing of adults with culling seropositive sheep or (ii) artificial rearing of lambs deprived of colostrum from infected dams, are expensive and have limited applicability in large production flocks (Houwers *et al*. 1983, 1984, 1989).

Breed differences in both SRLV seroprevalence and proviral concentration have suggested that genetics may play an important role in susceptibility (Gates *et al*. 1978; Cutlip *et al*. 1986; Houwers *et al*. 1989; Herrmann-Hoesing *et al*. 2008). Because SRLV induces lifelong infection, serological status has high concordance with direct viral measures of infection and can be used to measure odds of infection (Herrmann-Hoesing *et al*. 2007). Certain breeds have been associated consistently with higher or lower odds of infection. For example, Rambouillet has lower odds of infection than do other breeds (Gates *et al*. 1978; Cutlip *et al*. 1986; Houwers *et al*. 1989; Herrmann-Hoesing *et al*. 2008), and Columbia sheep have higher odds of infection (Herrmann-Hoesing *et al*. 2008). Proviral concentration significantly associates with severity of disease pathology, with high proviral concentration corresponding to high lesion score (Herrmann-Hoesing *et al*. 2009). Breed differences in level of proviral concentration are consistent with differences in odds of infection (Herrmann-Hoesing *et al*. 2008). Identifying genetic markers in specific breeds that associate with high proviral concentrations and removal or separation of these animals could result in reducing transmission and associated pathology of SRLV.

Recently, it has been discovered that variants in the *transmembrane protein 154* (*TMEM154*) gene were associated with odds of SRLV infection, and the association has been validated in multiple large animal sets (Heaton *et al*. 2012). Little is known about the function of *TMEM154* beyond transmembrane domain prediction, but its association with asthma severity in humans suggests a possible conserved role in airway immunity (Slager *et al*. 2011). Marker validation is important to reduce the probability of false-positive results and to improve reliability of predictive use (Hirschhorn *et al*. 2002; Li & Meyre 2013; White & Knowles 2013). *TMEM154* haplotypes with strong supporting data include haplotype 1 [containing lysine (K) at position 35] and haplotypes 2 and 3 [containing glutamic acid (E) at position 35]. Of these, sheep with two copies of haplotype 1 were less susceptible to SRLV infection, but sheep with at least one copy of either haplotypes 2 or 3 were more susceptible (Heaton *et al*. 2012). A standardized genotyping method has been developed and commercialized for sheep producers to take advantage of marker-assisted selection to reduce susceptibility (Heaton *et al*. 2013). In addition, it is possible that sheep homozygous for *TMEM154* haplotype 1 may also have lower proviral concentrations and lesion severity among infected sheep. However, sheep with these *TMEM154* haplotypes have not been examined for control of SRLV post-infection.

Additional opportunities exist for developing genetic markers connected with control of SRLV post-infection based on the ovine *chemokine (C-C motif) receptor 5* (*CCR5*) gene. Both SRLV and HIV are macrophage-tropic lentiviruses (Gendelman *et al*. 1986; Gorrell *et al*. 1992; Alkhatib & Berger 2007). Individual human beings with the delta-32 frame-shift deletion in *CCR5* show high natural resistance against HIV, and that resistance has been associated with lack of functional CCR5 protein on the cell surface (Kaslow *et al*. 2005; Alkhatib & Berger 2007). A more subtle relationship between SRLV and *CCR5* has been identified in sheep. Specifically, a four base-pair promoter deletion in ovine *CCR5* has been associated with lower SRLV proviral concentration; this promoter deletion was also associated with lower expression of *CCR5* (White *et al*. 2009). However, this association has been identified in only one flock, and it needs to be validated with additional flocks.

In the current study, we hypothesized that the previously defined low-risk *TMEM154* diplotype (two copies of haplotype 1) would be associated with lower SRLV proviral concentration in multiple flocks of sheep. Also, we hypothesized that a four base-pair deletion in the promoter of ovine *CCR5* would confirm an association with lower SRLV proviral concentration in multiple flocks of sheep. If either of these hypotheses were confirmed, then these data would provide evidence supporting one or more genetic markers for marker-assisted selection to achieve lower proviral concentration of SRLV.

## Materials and methods

### Phenotype and populations

Blood was collected by jugular venipuncture from four different flocks from three US states totaling 2236 ewes. Genomic DNA was extracted from peripheral blood leukocytes (PBLs) using previously described protocols (Herrmann-Hoesing *et al*. 2008; Heaton *et al*. 2012; White *et al*. 2012). In 2004, 353 ewes with a mean age of 4.35 years were sampled from an Idaho flock and included approximately equal numbers of Rambouillet, Polypay and Columbia breeds (Herrmann-Hoesing *et al*. 2008). A 2008 cohort from the same Idaho flock containing 947 ewes of Rambouillet, Polypay and Columbia breeds with a mean age of 2.42 years were also sampled (White *et al*. 2012). The average generation interval in the Idaho flock was approximately one every two to three years, and none of the animals were duplicated between the 2004 and 2008 Idaho sheep flocks. In 2009, 340 Polypay ewes with a mean age of 3.16 years were sampled from Iowa (Heaton *et al*. 2012), and 596 Rambouillet–Columbia crossbred ewes with a mean age of 3.18 years were sampled from Montana.

Proviral concentration of SRLV was determined by a validated qPCR method with over 95% concordance with serological methods (Herrmann-Hoesing *et al*. 2007). Briefly, PBLs were isolated from sheep blood, and DNA was extracted following the manufacturer's directions for Puregene (Genra System, Inc.). Real-time qPCR was performed using amplification primers for SRLV of a forward primer from the transmembrane gene, *tm* (5′-TCATAGTGCTTGCTATCATGGCTA-3′), and a reverse primer from the envelope glycoprotein gene, *env* (5′CCGTCCTTGTGTAGGATTGCT-3′). The SRLV *tm*-specific TaqMan probe, 5′-5′ hexachlorofluorescein-AGCAACACCGAGACCAGCTCCTGC-3′ Black Hole Quencher-1 (Integrated DNA Technologies) was used to quantify SRLV copy number (Herrmann-Hoesing *et al*. 2007). Up to 1 μg of DNA, 300 nm (final volume) of amplification primers, 250 nm of probe and TaqMan master mix diluted according to manufacturer's instructions (Applied Biosystems) were used in 50-μl reactions. Cycling conditions were 95 °C for 10 min followed by 60 cycles of 95 °C for 60 s and 55 °C for 60 s and 4 °C indefinitely for triplicate reactions (Herrmann-Hoesing *et al*. 2007). Known amounts (10^0^–10^7^ copies) of SRLV-containing plasmid were used in similar, triplicate reactions to generate standard curves. The SRLV copy numbers of unknown samples were determined using the mean threshold cycle value and the equation of the line generated in the standard curve (Herrmann-Hoesing *et al*. 2007). Proviral concentrations of zero were treated as SRLV negative for prevalence calculations.

### Sequence variants and genotyping

*TMEM154* genotypes were generated by Sanger sequencing of PCR fragments amplified from genomic DNA for 947 samples from Idaho 2008, 340 samples from Iowa, 353 samples from Idaho 2004 and 352 samples from Montana, as previously described (Heaton *et al*. 2012). Partway through the study, a faster and cheaper genotyping method became commercially available with greater than 98.5% concordance to the earlier PCR/sequencing method (Heaton *et al*. 2013). Therefore, another 244 samples from Montana were genotyped commercially by GeneSeek, Inc., using the new mass-spectrometric genotyping method (Heaton *et al*. 2013).

A deletion (g.52945778_52945781delATTC relative to accession NC_019476.1) in the promoter region of ovine *CCR5* was genotyped as previously described (White *et al*. 2009). Briefly, fluorescent TaqMan genotyping was performed per manufacturer's protocol (Applied Biosystems) as previously described (White *et al*. 2009). An earlier restriction fragment length polymorphism assay (White *et al*. 2009) was used as a supplementary genotyping approach in some samples either as a primary genotyping method for the oldest samples or for verification purposes on some later samples (Idaho 2004 and Iowa 2009).

### Statistical and bioinformatic analyses

phase 2.1 (Stephens *et al*. 2001; Stephens & Donnelly 2003) was used to determine *TMEM154* diplotypes for 352 ewes from the Montana flock that were genotyped by sequencing. The confounded genotypes from these sequenced animals were processed using phase-known settings with diplotypes from commercial genotyping of additional animals from the same flock (Stephens *et al*. 2001; Stephens & Donnelly 2003). Compliance with Hardy–Weinberg proportions was determined for all genotypes using chi-square tests prior to further statistical analysis.

**Figure 1:**
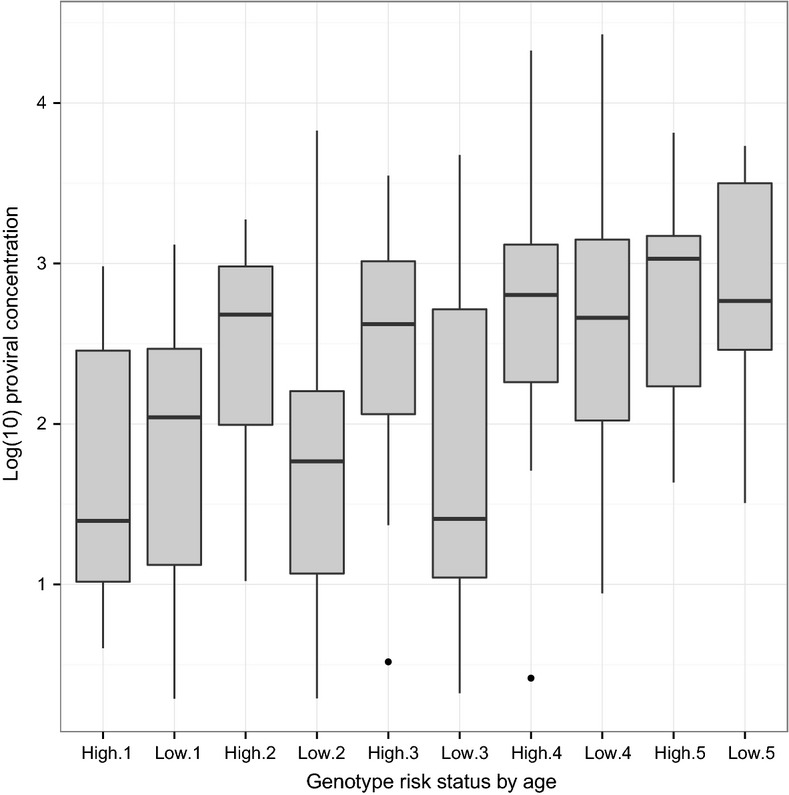
Boxplot of log_10_-transformed proviral concentration by age and *TMEM154* diplotype risk status in the Iowa flock.

Mean proviral concentrations shown in Fig. 1 were calculated by simple average of log_10_-transformed proviral concentrations that were then reverse-transformed to copies/μg DNA scale. The scale transformation was performed to reduce the influence of outlier individuals (extremely high proviral concentration) in calculating mean proviral concentration. The mixed model procedure of sas v9.2 (SAS Institute) was used to examine association of genotypic variants with log_10_-transformed proviral concentration among positive animals. Proviral concentration was the dependent variable, and breed, age and genotype were included as fixed effects in the association models. Genotypes of interest were defined as previously reported for the *CCR5* insertion/deletion (White *et al*. 2009). For *TMEM154*, only diplotypes 1,1, 1,2, 1,3, 2,2, 2,3 and 3,3 were included in analyses. Simultaneous testing was performed to analyze information content of *TMEM154* haplotypes and *CCR5* promoter deletion in one test, and the rest of the association model was as stated above. Furthermore, joint analysis of genotypes from all flocks was performed using the mixed model procedure of sas 9.2. Models were similar to those described above, but they also included random terms for location and year. All reported *P*-values were nominal and were not adjusted for multiple testing. The ggplot2 graphics package (Wickham 2009) in r v3.0.1 (Team 2011) was used for figure construction.

## Results

Only the 1403 SRLV-positive sheep from Idaho 2004, Idaho 2008, Iowa and Montana were included in association analysis for proviral concentration and *TMEM154* mutations or *CCR5* promoter deletion. Adjusted mean proviral concentrations and other summary data by flock are shown in Table 1. A significant association was identified between *TMEM154* and lower proviral concentration in the Iowa flock (*P* = 0.017; Table 2). Specifically, sheep with two copies of haplotype 1 had half the adjusted mean proviral concentration compared to sheep with one or more copies of haplotypes 2 or 3 (Table 2). The other three flocks had no identified association between *TMEM154* and proviral concentration (*P* > 0.05; Table 2); however, these flocks had very low haplotype frequencies for haplotypes 2 and 3 (Table 3). Specifically, one ewe in the Montana flock, 19 ewes in Idaho 2004 and 32 ewes in Idaho 2008 had haplotype 2 or 3 (Table 3). The nonsignificant results were: Idaho 2004 (*P* = 0.064) with adjusted mean proviral concentrations of 304 and 774 for diplotypes 1,1 and those containing haplotypes 2 or 3 respectively, Idaho 2008 (*P* = 0.63) with adjusted means of 123 and 102 respectively, and Montana was inestimable on an individual flock basis with mean proviral concentration of 1329 for 1,1 diplotypes. The joint analysis of all flocks was significant (*P* = 0.013; Table 2).

**Table 1:** Average proviral concentration from different flocks of sheep

Animal set	Montana	Idaho 2004	Idaho 2008	Iowa
Total animals genotyped	596	353	947	340
Year of sampling	2009	2004	2008	2009
Breeds included	Rambouillet–Columbia crossbred	Rambouillet, Polypay and Columbia	Rambouillet, Polypay and Columbia	Polypay
Proviral concentration	4770	1530	1250	1210
qPCR-positive sheep	607/620 = 97.9%	226/377 = 60.0%	368/947 = 38.9%	202/340 = 59.4%

**Table 2:** Association between *TMEM154* diplotypes and small ruminant lentivirus proviral concentration, including adjusted mean proviral concentration^1^ by diplotype

		Diplotype	
		
Flock	*P*-value	1,1	Contain 2 or 3
Iowa	0.017	131	270
Idaho 2004	NS^2^		
Idaho 2008	NS^2^		
Montana	NS^2^		
All flocks	0.013	311	508

1 Adjusted means were reverse-transformed to the copies/μg DNA scale.

2 Not significant (*P* > 0.05)

**Table 3:** Number of small ruminant lentivirus infected individuals bearing different *TMEM154* diplotypes by flock

Flock	Montana	Idaho 2004	Idaho 2008	Iowa	Total
Diplotype 1,1	595	171	333	88	1187
Diplotypes containing 2 or 3	1	19	32	94	146
Total animals	596	190	365	182	1333

A significant association was identified between the *CCR5* promoter insertion and proviral concentration in the Iowa flock (*P* < 0.05; Table 4). The adjusted mean log_10_ proviral concentration was higher in the deletion homozygotes than in the insertion homozygotes and heterozygotes (Table 4). The Montana and Idaho 2008 sheep flocks showed no significant association between *CCR5* promoter deletion and proviral concentration (*P* > 0.05; Table 4). Specifically, for Montana, the association significance was *P* = 0.55 with adjusted means 1202, 1479 and 1288 for insertion homozygote, heterozygote and deletion homozygote respectively. For Idaho 2008, the association was significance was *P* = 0.96 with adjusted means of 120, 115 and 107 respectively. Numbers of animals by *CCR5* genotype are shown in Table S1. The joint analysis of *CCR5* including all flocks was significant (*P* = 0.028; Table 4). Simultaneous testing of markers in both genes was significant in the joint all-flocks analysis: *TMEM154* (*P* = 0.016) and *CCR5* (*P* = 0.023). Individual flock analyses provided similar results to the individual gene, single flock analyses: *TMEM154* (*P* = 0.023) and *CCR5* (*P* = 0.011) in the Iowa flock, *TMEM154* (*P* > 0.05) and *CCR5* (*P* = 0.010) for Idaho 2004 and (*P* > 0.05) for both genes in the other flocks. The breed, proviral concentration, *TMEM154* diplotypes, age and *CCR5* promoter variant genotypes for all animals in this study are shown in Table S2.

**Table 4:** Association between *CCR5* promoter deletion and small ruminant lentivirus proviral concentration, including adjusted mean proviral concentration^1^ by genotype

		Genotype^2^		
		
Flock	*P*-value	II	ID	DD
Iowa	0.041	126	288	371
Idaho 2004^3^	0.0077^3^	312^3^	494^3^	111^3^
Idaho 2008	NS^4^			
Montana	NS^4^			
All flocks	0.028	280	377	270

1 Adjusted means were reverse-transformed to the copies/μg DNA scale.

2 DD is homozygous deletion, II is homozygous insertion, and ID is heterozygous insertion/deletion.

3 Data from White *et al*. (2009).

4 Not significant (*P* > 0.05).

## Discussion

This study examined the association between specific variants in two genes and SRLV proviral concentration using multiple flocks of sheep. Markers in the first gene, *TMEM154*, had already been validated for SRLV odds of infection but had never been examined for any measure of control post-infection. A marker in the second gene, *CCR5*, was associated with proviral concentration in one flock. If either variant was consistently associated with proviral concentration in multiple flocks of sheep, it would support potential use of one or more validated genetic markers for post-infection control of SRLV in sheep.

Sheep with two copies of *TMEM154* haplotype 1 had half the proviral concentration compared with those with at least one copy of haplotype 2 or 3 in the Iowa flock (Table 2). Thus, the same diplotype previously identified with lower risk of initial SRLV infection (Heaton *et al*. 2012) also had improved control of SRLV post-infection in the Iowa flock. Boxplots by age and *TMEM154* risk status are shown in Fig. 1. Only ages one to five years were included due to very low numbers of sheep age six to eight years. Almost all ages showed higher median proviral concentration in high-risk compared with low-risk diplotypes; the only exception was one year of age, when the high-risk diplotypes had lower median proviral concentration. Ages two and three years show the largest differences in median proviral concentration. At more advanced ages, smaller numbers of ewes with *TMEM154* high-risk haplotype 2 or haplotype 3 could be due to culling or premature death (Fig. 1). Further, haplotype 1 appears to have a recessive mode of inheritance because two copies of haplotype 1 were required to show lower proviral concentration, which was also true for lower odds of infection (Heaton *et al*. 2012).

Although the joint analysis of all flocks showed association between *TMEM154* diplotypes and SRLV proviral concentration (*P* = 0.013; Table 2), this analysis was dominated by the Iowa flock. The Idaho 2004, Idaho 2008 and Montana sheep flocks did not show significant association between *TMEM154* haplotypes and proviral concentration in individual flock analysis (Table 2). However, the lack of association may be due to low frequencies of haplotypes 2 and 3 in these three sheep flocks (Table 3). None of these flocks had more than 32 individuals with haplotypes 2 or 3, compared with the Iowa flock, which had 94 ewes with haplotype 2 and/or 3. Other flocks with similar breed composition have been found to have higher allele frequencies of haplotype 2 and 3 (Heaton *et al*. 2013), and the low frequencies here are consistent with a hypothesis of historical selection against haplotypes 2 and 3 in these flocks that had a high occurrence of SRLV infection. It is also possible that the different allele frequencies between flocks could be due to genetic drift, as from founder effects. Without access to historical samples, it is not possible at present to distinguish selection from other potential explanations. In order to validate the association between *TMEM154* haplotypes and proviral concentration in the Iowa flock, additional sheep flocks with higher frequencies of haplotype 2 and/or haplotype 3 among SRLV infected sheep will need to be identified and tested. If this genetic marker is validated for SRLV proviral concentration, it can be used for marker-assisted selection to not only reduce susceptibility but also to lower proviral concentration and severity of disease.

Previously, the *CCR5* promoter deletion homozygotes were associated with lower proviral concentration in one flock (Idaho 2004) (White *et al*. 2009). Here, the Iowa animal set showed significant association with genotypes at this locus, but the direction of the association was opposite compared with previous findings (Table 4). Insertion homozygotes had lower proviral concentration than either deletion homozygotes or insertion/deletion heterozygotes. Although the joint analysis of all flocks was significant (*P* = 0.028; Table 4), the genotypic adjusted means showed higher proviral concentration in heterozygotes than in either homozygote. This can be explained by the opposite directions of association between the Idaho 2004 and Iowa flocks. In each case, one homozygote and the heterozygote showed high proviral concentration. When considered in an overall joint analysis, only the heterozygote was consistently associated with high proviral concentration. Further, current data showed no significant association in the other sheep flocks, even with testable frequencies for all genotypes (Table S1). Clearly, there was no consistent association between the *CCR5* deletion and control of SRLV post-infection (Table 4).

There are multiple possible explanations for the inconsistent association of the *CCR5* promoter deletion and SRLV proviral concentration. One possible reason is that the *CCR5* promoter variant might occur in differing degrees of linkage disequilibrium with one or more additional underlying functional variants that are important for control of SRLV in different sheep flocks (Thormar 2005; Li & Meyre 2013), despite gene expression differences noted previously (White *et al*. 2009). Because CCR5 functions in signaling pathways that promote chemotaxis, lower expression of *CCR5* may reduce chemotaxis of macrophages and other target leukocytes toward infected cells and slow cellular spread (Locati *et al*. 2002; Ma *et al*. 2005). Alternatively, a second explanation for the differing directions of association might occur because of one or more virus subtypes. Retroviruses mutate rapidly, and it is possible that some subtypes adapted to the insertion allele instead of the deletion allele. A third possibility could be unidentified co-infections with other, as yet unknown, immunomodulatory pathogens (Walson & John-Stewart 2007). Co-infection with such pathogens could change immune responses and SRLV disease severity in some sheep populations but not others where such co-infections were absent and, thus, change the direction of genetic variant association. Therefore, additional studies are necessary to clarify the role of the *CCR5* promoter deletion in SRLV infection of sheep and in the immune system more generally.

## Summary

In conclusion, testing for association between control of SRLV post-infection and genotypes for *TMEM154* and *CCR5* variants suggests different approaches to further work in each case. This is the first report of an association between *TMEM154* diplotypes and SRLV proviral concentration, and the same desirable *TMEM154* haplotypes associated with decreased proviral concentration in this flock were also associated with decreased SRLV odds of infection in a previous report (Heaton *et al*. 2012). Future work with additional sheep flocks possessing more balanced diplotype frequencies is needed to confirm or refute the association of *TMEM154* with SRLV proviral concentration. For *CCR5*, a promoter deletion was not consistently associated with proviral concentration in multiple flocks of sheep. Further work with additional variants in the genomic region, SRLV strain data and/or co-infection data concerning other pathogens may help explain the complicated patterns of association observed here.

## Supporting information

**Table S1.** Numbers of positive individuals by *CCR5* genotype from four flocks.

**Table S2.** All animals included in the study with breed, mean qPCR, age, *CCR5* genotype and *TMEM154* diplotype.
