# Supplementary material for: Mutations in Ovis aries TMEM154 are associated with lower small ruminant lentivirus proviral concentration in one sheep flock
Source: Anim Genet. 2014 Jun 17;45(4):565–71. doi: 10.1111/age.12181 (PMC4140605; doi:10.1111/age.12181)
Supplement: Supplementary file 1 — Table S1. Numbers of positive individuals by CCR5 genotype from four flocks. [file age0045-0565-SD1.pdf]

Table S1: Numbers of positive individuals by *CCR5* genotype from four flocks.

**Flock**	Montana	Idaho 2008	Iowa	Total
**DD**[1]	142	27	12	181
**II**[1]	197	168	114	479
**ID**[1]	317	135	75	527
**Total**	656	330	201	1187

[1]DD is homozygous deletion, II is homozygous insertion and ID is heterozygous insertion/deletion
