# Supplementary material for: Mutations in Ovis aries TMEM154 are associated with lower small ruminant lentivirus proviral concentration in one sheep flock
Source: Anim Genet. 2014 Jun 17;45(4):565–71. doi: 10.1111/age.12181 (PMC4140605; doi:10.1111/age.12181)
Supplement: Supplementary file 2 — Table S2. All animals included in the study with breed, mean qPCR, age, CCR5 genotype and TMEM154 diplotype. [file age0045-0565-SD2.pdf]

Table S2
All animals included in the study with breed, mean qPCR, age, CCR5 genotype and TMEM154 diplotype

Animal Number	Breed	Flock region	age	Mean qPCR	TMEM154 Diplotype	CCR5 Genotype
**1**	Polypay	Iowa	4	245	13	II
**2**	Polypay	Iowa	3	.	11	ID
**3**	Polypay	Iowa	4	.	11	II
**4**	Polypay	Iowa	3	125	13	ID
**5**	Polypay	Iowa	3	63	13	ID
**6**	Polypay	Iowa	3	825	13	ID
**7**	Polypay	Iowa	3	.	11	II
**8**	Polypay	Iowa	3	8.97	11	II
**9**	Polypay	Iowa	3	3..4	12	II
**10**	Polypay	Iowa	3	.	11	II
**11**	Polypay	Iowa	3	19.6	11	II
**12**	Polypay	Iowa	5	.	11	II
**13**	Polypay	Iowa	1	14.7	12	ID
**14**	Polypay	Iowa	4	.	11	DD
**15**	Polypay	Iowa	4	88.6	13	ID
**16**	Polypay	Iowa	5	.	11	ID
**17**	Polypay	Iowa	5	.	11	ID
**18**	Polypay	Iowa	3	11	13	ID
**19**	Polypay	Iowa	7	179	11	ID
**20**	Polypay	Iowa	4	.	11	II
**21**	Polypay	Iowa		.	11	II
**22**	Polypay	Iowa	3	.	11	II
**23**	Polypay	Iowa	3	6.4	11	II
**24**	Polypay	Iowa	3	94.4	11	II
**25**	Polypay	Iowa	6	599	11	ID
**26**	Polypay	Iowa	3	571	13	II
**27**	Polypay	Iowa	5	42.2	11	II
**28**	Polypay	Iowa	4	14.1	11	II
**29**	Polypay	Iowa	3	.	11	II
**30**	Polypay	Iowa	4	111	11	II
**31**	Polypay	Iowa	4	829	12	ID
**32**	Polypay	Iowa	5	.	11	DD
**33**	Polypay	Iowa	3	.	11	ID
**34**	Polypay	Iowa		2.22	11	DD
**35**	Polypay	Iowa	5	86	11	II
**36**	Polypay	Iowa	4	356	11	ID
**37**	Polypay	Iowa	4	146	11	DD

**38**	Polypay	Iowa	3	235	13	II
**39**	Polypay	Iowa	4	133	13	II
**40**	Polypay	Iowa	3	29.8	12	II
**41**	Polypay	Iowa	4	.	11	ID
**42**	Polypay	Iowa	3	.	11	II
**43**	Polypay	Iowa	5	14	23	II
**44**	Polypay	Iowa	3	.	11	II
**45**	Polypay	Iowa	3	23.4	13	ID
**46**	Polypay	Iowa	3	.	11	II
**47**	Polypay	Iowa	1	131	11	DD
**48**	Polypay	Iowa	3	113	12	II
**49**	Polypay	Iowa	3	.	11	II
**50**	Polypay	Iowa	3	61.2	13	II
**51**	Polypay	Iowa	4	136	11	ID
**52**	Polypay	Iowa	3	.		II
**53**	Polypay	Iowa	3	.		II
**54**	Polypay	Iowa	3	333		II
**55**	Polypay	Iowa	5	.	11	DD
**56**	Polypay	Iowa	3	49	13	II
**57**	Polypay	Iowa	4	.	11	II
**58**	Polypay	Iowa	6	1.4.	33	ID
**59**	Polypay	Iowa	3	.	11	II
**60**	Polypay	Iowa	5	584	11	II
**61**	Polypay	Iowa	3	265	12	ID
**62**	Polypay	Iowa		677		DD
**63**	Polypay	Iowa	5	65		DD
**64**	Polypay	Iowa	4	193	11	ID
**65**	Polypay	Iowa	3	3.3	13	ID
**66**	Polypay	Iowa	4	.	11	ID
**67**	Polypay	Iowa	5	142	13	II
**68**	Polypay	Iowa	5	21	33	II
**69**	Polypay	Iowa	5	323	11	II
**70**	Polypay	Iowa	4	99.4	11	II
**71**	Polypay	Iowa	4	.	11	ID
**72**	Polypay	Iowa	1	.	11	ID
**73**	Polypay	Iowa	4	.	11	DD
**74**	Polypay	Iowa	3	358	13	ID
**75**	Polypay	Iowa	4	868	11	DD
**76**	Polypay	Iowa	1	.	11	ID
**77**	Polypay	Iowa	3	.	11	II
**78**	Polypay	Iowa	4	.	12	II
**79**	Polypay	Iowa	3	16.6		ID
**80**	Polypay	Iowa	4	.	11	ID

**81**	Polypay	Iowa	3	32.2	11	II
**82**	Polypay	Iowa	3	61.4	12	ID
**83**	Polypay	Iowa	3	119	11	ID
**84**	Polypay	Iowa	3	.	11	II
**85**	Polypay	Iowa	4	474	13	ID
**86**	Polypay	Iowa	4	43.6	11	II
**87**	Polypay	Iowa	2	1.95	11	II
**88**	Polypay	Iowa	2	.	11	ID
**89**	Polypay	Iowa	4	.	13	ID
**90**	Polypay	Iowa	2	.	11	II
**91**	Polypay	Iowa	3	.	11	II
**92**	Polypay	Iowa	1	.	11	II
**93**	Polypay	Iowa	4	126	13	ID
**94**	Polypay	Iowa	4	117	13	II
**95**	Polypay	Iowa	4	562	11	II
**96**	Polypay	Iowa	3	.	11	II
**97**	Polypay	Iowa	4	415	12	ID
**98**	Polypay	Iowa	2	6.58	11	II
**99**	Polypay	Iowa	4	192	13	II
**100**	Polypay	Iowa	2	.	11	II
**101**	Polypay	Iowa	1	153	13	II
**102**	Polypay	Iowa	1	.	11	II
**103**	Polypay	Iowa	4	.	11	ID
**104**	Polypay	Iowa	1	.	11	II
**105**	Polypay	Iowa	1	4..1	12	II
**106**	Polypay	Iowa	1	.	12	II
**107**	Polypay	Iowa	1	.	11	II
**108**	Polypay	Iowa	1	867	11	ID
**109**	Polypay	Iowa	4	268	13	II
**110**	Polypay	Iowa	1	39.3	13	II
**111**	Polypay	Iowa	1	2.28	11	II
**112**	Polypay	Iowa	3	.	11	ID
**113**	Polypay	Iowa	4	.	11	ID
**114**	Polypay	Iowa	3	491	12	II
**115**	Polypay	Iowa	1	.	13	ID
**116**	Polypay	Iowa	1	.	11	II
**117**	Polypay	Iowa	1	353	12	ID
**118**	Polypay	Iowa	1	15.8	12	II
**119**	Polypay	Iowa	5	155	13	II
**120**	Polypay	Iowa	1	96	13	II
**121**	Polypay	Iowa	1	.	13	II
**122**	Polypay	Iowa	3	352	13	II
**123**	Polypay	Iowa	1	.	11	II

**124**	Polypay	Iowa	1	116	11	II
**125**	Polypay	Iowa	1	.	11	II
**126**	Polypay	Iowa	1	.	11	II
**127**	Polypay	Iowa	1	.	11	II
**128**	Polypay	Iowa	1	384	12	ID
**129**	Polypay	Iowa	2	.	11	ID
**130**	Polypay	Iowa	3	.	11	II
**131**	Polypay	Iowa	3	1.4.	13	ID
**132**	Polypay	Iowa	2	112	12	II
**133**	Polypay	Iowa	3	.	11	II
**134**	Polypay	Iowa	2	.	11	II
**135**	Polypay	Iowa	2	.	11	DD
**136**	Polypay	Iowa	2	.	11	II
**137**	Polypay	Iowa	3	474	11	II
**138**	Polypay	Iowa	4	.	11	DD
**139**	Polypay	Iowa	4	.	11	II
**140**	Polypay	Iowa	3	132	13	II
**141**	Polypay	Iowa	3	.	11	II
**142**	Polypay	Iowa	2	26	11	II
**143**	Polypay	Iowa	4	.	11	DD
**144**	Polypay	Iowa	4	121	13	ID
**145**	Polypay	Iowa	2	1..5	13	II
**146**	Polypay	Iowa	3	563	11	II
**147**	Polypay	Iowa	1	.	11	II
**148**	Polypay	Iowa	1	.		II
**149**	Polypay	Iowa		.	11	ID
**150**	Polypay	Iowa	3	.		DD
**151**	Polypay	Iowa	2	.		II
**152**	Polypay	Iowa	1	.	12	ID
**153**	Polypay	Iowa	7	23.3	33	DD
**154**	Polypay	Iowa	2	98.7	12	II
**155**	Polypay	Iowa	6	.		DD
**156**	Polypay	Iowa	3	95..		II
**157**	Polypay	Iowa	1	.		II
**158**	Polypay	Iowa	2	673	11	ID
**159**	Polypay	Iowa	3	634	11	II
**160**	Polypay	Iowa	1	6.97	13	II
**161**	Polypay	Iowa	2	.	11	II
**162**	Polypay	Iowa	2	.	11	II
**163**	Polypay	Iowa	3	221	11	ID
**164**	Polypay	Iowa	3	.	11	II
**165**	Polypay	Iowa	1	82.9		ID
**166**	Polypay	Iowa	2	152		II

**167**	Polypay	Iowa	2	.	11	ID
**168**	Polypay	Iowa	2	371	13	II
**169**	Polypay	Iowa	3	.	11	II
**170**	Polypay	Iowa	2	.	11	II
**171**	Polypay	Iowa	2	188	12	II
**172**	Polypay	Iowa	3	12.8	11	ID
**173**	Polypay	Iowa	1	1.4	11	ID
**174**	Polypay	Iowa	4	51.2	13	II
**175**	Polypay	Iowa	3	14..		ID
**176**	Polypay	Iowa	1	.	11	ID
**177**	Polypay	Iowa	1	.	11	II
**178**	Polypay	Iowa	2	.	11	ID
**179**	Polypay	Iowa	4	356	11	ID
**180**	Polypay	Iowa	3	791	33	II
**181**	Polypay	Iowa	2	927		ID
**182**	Polypay	Iowa	3	.		ID
**183**	Polypay	Iowa	3	117	12	ID
**184**	Polypay	Iowa	4	111	23	II
**185**	Polypay	Iowa	4	131	13	II
**186**	Polypay	Iowa	2	1.4	11	II
**187**	Polypay	Iowa	5	.	11	DD
**188**	Polypay	Iowa	2	159	13	II
**189**	Polypay	Iowa	4	.		DD
**190**	Polypay	Iowa	2	43.7	12	II
**191**	Polypay	Iowa	5	29	11	II
**192**	Polypay	Iowa	5	54..	11	II
**193**	Polypay	Iowa	8	9.24	13	II
**194**	Polypay	Iowa		.	11	ID
**195**	Polypay	Iowa	2	486	13	ID
**196**	Polypay	Iowa	3	188	12	II
**197**	Polypay	Iowa	4	.	11	ID
**198**	Polypay	Iowa	2	.	11	ID
**199**	Polypay	Iowa	6	2.13	11	II
**200**	Polypay	Iowa	5	.	11	ID
**201**	Polypay	Iowa	5	316	11	II
**202**	Polypay	Iowa	4	231	13	ID
**203**	Polypay	Iowa	3	.	11	ID
**204**	Polypay	Iowa	3	1.3.	13	II
**205**	Polypay	Iowa	3	.	11	ID
**206**	Polypay	Iowa	1	9.26	13	II
**207**	Polypay	Iowa	3	2.1	11	ID
**208**	Polypay	Iowa	1	.	11	II
**209**	Polypay	Iowa	1	.	11	II

**210**	Polypay	Iowa	2	.	12	II
**211**	Polypay	Iowa	4	8.81	11	ID
**212**	Polypay	Iowa	4	319	12	ID
**213**	Polypay	Iowa	2	183	11	II
**214**	Polypay	Iowa	1	.	11	II
**215**	Polypay	Iowa	1	.	11	ID
**216**	Polypay	Iowa	3	2.9	11	II
**217**	Polypay	Iowa	4	.	11	II
**218**	Polypay	Iowa	2	.	11	II
**219**	Polypay	Iowa	4	521	12	II
**220**	Polypay	Iowa	4	.	11	II
**221**	Polypay	Iowa	5	223	11	ID
**222**	Polypay	Iowa	4	141		DD
**223**	Polypay	Iowa	3	.	11	ID
**224**	Polypay	Iowa	3	324	13	ID
**225**	Polypay	Iowa	4	147	13	II
**226**	Polypay	Iowa	4	889	11	II
**227**	Polypay	Iowa	4	125	13	II
**228**	Polypay	Iowa	3	355	12	II
**229**	Polypay	Iowa	4	131	11	II
**230**	Polypay	Iowa	2	.	11	II
**231**	Polypay	Iowa	8	22...	11	ID
**232**	Polypay	Iowa	4	.	11	II
**233**	Polypay	Iowa	5	651	12	ID
**234**	Polypay	Iowa	4	313	11	ID
**235**	Polypay	Iowa		.	11	ID
**236**	Polypay	Iowa	1	.	11	II
**237**	Polypay	Iowa	3	4.28	11	II
**238**	Polypay	Iowa		318	11	II
**239**	Polypay	Iowa	5	43.2	13	II
**240**	Polypay	Iowa	2	48	13	ID
**241**	Polypay	Iowa	3	142	12	II
**242**	Polypay	Iowa	5	213		II
**243**	Polypay	Iowa	8	6.76	11	II
**244**	Polypay	Iowa	5	.	11	DD
**245**	Polypay	Iowa	2	16	11	II
**246**	Polypay	Iowa	2	.	11	ID
**247**	Polypay	Iowa	3	.	11	DD
**248**	Polypay	Iowa	4	.	11	II
**249**	Polypay	Iowa	4	397	12	II
**250**	Polypay	Iowa	4	226	11	ID
**251**	Polypay	Iowa	4	69	11	ID
**252**	Polypay	Iowa	3	.	11	II

**253**	Polypay	Iowa		136		ID
**254**	Polypay	Iowa	4	24..	11	ID
**255**	Polypay	Iowa	3	.	11	ID
**256**	Polypay	Iowa	3	.	11	II
**257**	Polypay	Iowa	3	162	13	ID
**258**	Polypay	Iowa		356	13	II
**259**	Polypay	Iowa	1	.	11	II
**260**	Polypay	Iowa	3	18.6		ID
**261**	Polypay	Iowa	1	.		ID
**262**	Polypay	Iowa	2	.	11	ID
**263**	Polypay	Iowa	3	.	12	II
**264**	Polypay	Iowa	3	19.9	14	II
**265**	Polypay	Iowa	2	.	11	ID
**266**	Polypay	Iowa	2	.	11	II
**267**	Polypay	Iowa	1	23.8	11	II
**268**	Polypay	Iowa	2	.	11	II
**269**	Polypay	Iowa	1	2.5	11	ID
**270**	Polypay	Iowa	6	23.2	11	II
**271**	Polypay	Iowa	2	.	11	II
**272**	Polypay	Iowa	4	373	13	ID
**273**	Polypay	Iowa	4	212..	33	ID
**274**	Polypay	Iowa	3	2.5	13	II
**275**	Polypay	Iowa	2	958	13	II
**276**	Polypay	Iowa	2	58.4	11	II
**277**	Polypay	Iowa	5	.		ID
**278**	Polypay	Iowa	4	18	11	II
**279**	Polypay	Iowa	4	52.4	13	II
**280**	Polypay	Iowa	4	27.1	11	II
**281**	Polypay	Iowa	4	267..	11	ID
**282**	Polypay	Iowa	6	147	11	II
**283**	Polypay	Iowa	3	.	11	ID
**284**	Polypay	Iowa	4	276	11	DD
**285**	Polypay	Iowa	4	.	11	II
**286**	Polypay	Iowa	3	.	11	II
**287**	Polypay	Iowa	3		13	ID
**288**	Polypay	Iowa	3	1..5	11	ID
**289**	Polypay	Iowa	3	.	11	II
**290**	Polypay	Iowa	4	62	11	ID
**291**	Polypay	Iowa		45.4	11	II
**292**	Polypay	Iowa	5	1.7.	13	ID
**293**	Polypay	Iowa	5	123	11	ID
**294**	Polypay	Iowa	3	599	13	ID
**295**	Polypay	Iowa	4	69.9	13	II

**296**	Polypay	Iowa	3	174	13	DD
**297**	Polypay	Iowa	4	221	11	ID
**298**	Polypay	Iowa	4	.	13	II
**299**	Polypay	Iowa	3	112	11	II
**300**	Polypay	Iowa	3	.	13	ID
**301**	Polypay	Iowa	3	41.8	11	ID
**302**	Polypay	Iowa	2	.	11	II
**303**	Polypay	Iowa	4	2.61	23	ID
**304**	Polypay	Iowa	3	972	12	II
**305**	Polypay	Iowa	4	459	11	DD
**306**	Polypay	Iowa	3	17.1	11	II
**307**	Polypay	Iowa	3	.	11	ID
**308**	Polypay	Iowa	2	266	12	II
**309**	Polypay	Iowa	3	5.46	11	II
**310**	Polypay	Iowa	1	1.94	11	II
**311**	Polypay	Iowa	4	.	11	II
**312**	Polypay	Iowa	3	19.5	11	II
**313**	Polypay	Iowa	3	.		DD
**314**	Polypay	Iowa	3	2..4	11	II
**315**	Polypay	Iowa	4	7..2	11	II
**316**	Polypay	Iowa	3	.	13	ID
**317**	Polypay	Iowa	5	32.2	11	II
**318**	Polypay	Iowa	3		13	ID
**319**	Polypay	Iowa	6	2.9	11	ID
**320**	Polypay	Iowa	4	637	13	ID
**321**	Polypay	Iowa		.	11	ID
**322**	Polypay	Iowa	5	441	11	ID
**323**	Polypay	Iowa	4	182	12	DD
**324**	Polypay	Iowa	3	.		ID
**325**	Polypay	Iowa	1	.	11	ID
**326**	Polypay	Iowa		89	11	II
**327**	Polypay	Iowa	5	331	11	ID
**328**	Polypay	Iowa	5	417	11	ID
**329**	Polypay	Iowa	3	.	11	II
**330**	Polypay	Iowa	4	313	23	II
**331**	Polypay	Iowa	3	4.5	11	II
**332**	Polypay	Iowa	4	575	12	ID
**333**	Polypay	Iowa	4	126	11	II
**334**	Polypay	Iowa	3	.	11	ID
**335**	Polypay	Iowa	2	758	13	ID
**336**	Polypay	Iowa	2	11.7	11	ID
**337**	Polypay	Iowa	3	.	11	II
**338**	Polypay	Iowa	3	.		ID

**339**	Polypay	Iowa	8	1.4	11	II
**340**	Polypay	Iowa	2	84.2	13	ID
**341**	Columbia-Rambouillet	Montana	6	166000	11	II
**342**	Columbia-Rambouillet	Montana	5	163000	11	ID
**343**	Columbia-Rambouillet	Montana	2	120000	11	DD
**344**	Columbia-Rambouillet	Montana	6	90700	11	DD
**345**	Columbia-Rambouillet	Montana	4	87300	11	ID
**346**	Columbia-Rambouillet	Montana	3	84800	11	DD
**347**	Columbia-Rambouillet	Montana	6	75900	11	DD
**348**	Columbia-Rambouillet	Montana	3	69300	11	II
**349**	Columbia-Rambouillet	Montana	3	68600	11	II
**350**	Columbia-Rambouillet	Montana	3	66700	11	ID
**351**	Columbia-Rambouillet	Montana	2	66100	11	ID
**352**	Columbia-Rambouillet	Montana	6	56500	11	ID
**353**	Columbia-Rambouillet	Montana	3	50700	11	DD
**354**	Columbia-Rambouillet	Montana	3	46500	11	II
**355**	Columbia-Rambouillet	Montana	4	43500	11	DD
**356**	Columbia-Rambouillet	Montana	3	42100	11	ID
**357**	Columbia-Rambouillet	Montana	4	39600	11	ID
**358**	Columbia-Rambouillet	Montana	3	39200	11	ID
**359**	Columbia-Rambouillet	Montana	8	39000	11	ID
**360**	Columbia-Rambouillet	Montana	5	38200	11	DD
**361**	Columbia-Rambouillet	Montana	6	37800	11	ID
**362**	Columbia-Rambouillet	Montana	4	36900	11	ID
**363**	Columbia-Rambouillet	Montana	4	35300	11	ID
**364**	Columbia-Rambouillet	Montana	5	35000	11	DD
**365**	Columbia-Rambouillet	Montana	2	34700	11	ID
**366**	Columbia-Rambouillet	Montana	5	32500	11	ID
**367**	Columbia-Rambouillet	Montana	2	32400	11	ID
**368**	Columbia-Rambouillet	Montana	6	31200	11	II
**369**	Columbia-Rambouillet	Montana	3	30700	11	ID
**370**	Columbia-Rambouillet	Montana	2	30300	11	II
**371**	Columbia-Rambouillet	Montana	3	29400	11	DD
**372**	Columbia-Rambouillet	Montana	6	28000	11	DD
**373**	Columbia-Rambouillet	Montana	6	26900	11	DD
**374**	Columbia-Rambouillet	Montana	3	23400	11	ID
**375**	Columbia-Rambouillet	Montana	5	23300	11	ID
**376**	Columbia-Rambouillet	Montana	7	22500	11	II
**377**	Columbia-Rambouillet	Montana	3	22200	11	II
**378**	Columbia-Rambouillet	Montana	2	21800	11	ID

**379**	Columbia-Rambouillet	Montana	2	19700	11	II
**380**	Columbia-Rambouillet	Montana	3	19700	11	II
**381**	Columbia-Rambouillet	Montana	.	18400	11	ID
**382**	Columbia-Rambouillet	Montana	3	18100	11	ID
**383**	Columbia-Rambouillet	Montana	3	17200	11	II
**384**	Columbia-Rambouillet	Montana	2	17000	11	II
**385**	Columbia-Rambouillet	Montana	2	16600	11	ID
**386**	Columbia-Rambouillet	Montana	3	15400	11	ID
**387**	Columbia-Rambouillet	Montana	4	14700	11	ID
**388**	Columbia-Rambouillet	Montana	7	14500	11	II
**389**	Columbia-Rambouillet	Montana	3	14500	11	DD
**390**	Columbia-Rambouillet	Montana	5	14300	11	ID
**391**	Columbia-Rambouillet	Montana	1	14200	11	II
**392**	Columbia-Rambouillet	Montana	6	13700	11	II
**393**	Columbia-Rambouillet	Montana	3	13600	11	ID
**394**	Columbia-Rambouillet	Montana	3	13500	11	ID
**395**	Columbia-Rambouillet	Montana	8	13500	11	II
**396**	Columbia-Rambouillet	Montana	5	13300	11	II
**397**	Columbia-Rambouillet	Montana	6	13000	11	ID
**398**	Columbia-Rambouillet	Montana	3	12900	11	ID
**399**	Columbia-Rambouillet	Montana	4	12700	11	DD
**400**	Columbia-Rambouillet	Montana	2	12300	11	ID
**401**	Columbia-Rambouillet	Montana	4	12000	11	DD
**402**	Columbia-Rambouillet	Montana	6	11900	11	ID
**403**	Columbia-Rambouillet	Montana	3	11700	11	ID
**404**	Columbia-Rambouillet	Montana	1	11700	11	ID
**405**	Columbia-Rambouillet	Montana	5	11400	11	ID
**406**	Columbia-Rambouillet	Montana	1	11300	11	II
**407**	Columbia-Rambouillet	Montana	.	11200	11	.
**408**	Columbia-Rambouillet	Montana	5	11100	11	DD
**409**	Columbia-Rambouillet	Montana	4	10600	11	II
**410**	Columbia-Rambouillet	Montana	2	10600	11	ID
**411**	Columbia-Rambouillet	Montana	5	10400	11	II
**412**	Columbia-Rambouillet	Montana	7	10300	11	ID
**413**	Columbia-Rambouillet	Montana	6	9910	11	II
**414**	Columbia-Rambouillet	Montana	5	9620	11	ID
**415**	Columbia-Rambouillet	Montana	7	9100	11	DD
**416**	Columbia-Rambouillet	Montana	2	9100	11	ID
**417**	Columbia-Rambouillet	Montana	4	9040	11	DD
**418**	Columbia-Rambouillet	Montana	2	8520	11	ID
**419**	Columbia-Rambouillet	Montana	4	8520	11	DD
**420**	Columbia-Rambouillet	Montana	1	8080	11	ID
**421**	Columbia-Rambouillet	Montana	6	8020	11	DD

**422**	Columbia-Rambouillet	Montana	5	7920	11	II
**423**	Columbia-Rambouillet	Montana	4	7910	11	II
**424**	Columbia-Rambouillet	Montana	4	7910	11	ID
**425**	Columbia-Rambouillet	Montana	.	7880	11	II
**426**	Columbia-Rambouillet	Montana	6	7650	11	ID
**427**	Columbia-Rambouillet	Montana	1	7600	11	ID
**428**	Columbia-Rambouillet	Montana	3	7250	11	DD
**429**	Columbia-Rambouillet	Montana	3	7200	11	ID
**430**	Columbia-Rambouillet	Montana	.	7130	11	II
**431**	Columbia-Rambouillet	Montana	1	7000	11	II
**432**	Columbia-Rambouillet	Montana	.	7000	11	ID
**433**	Columbia-Rambouillet	Montana	6	6840	11	II
**434**	Columbia-Rambouillet	Montana	1	6800	11	II
**435**	Columbia-Rambouillet	Montana	1	6500	11	ID
**436**	Columbia-Rambouillet	Montana	7	6470	11	ID
**437**	Columbia-Rambouillet	Montana	5	6400	11	DD
**438**	Columbia-Rambouillet	Montana	2	6380	11	ID
**439**	Columbia-Rambouillet	Montana	1	6280	11	ID
**440**	Columbia-Rambouillet	Montana	2	5900	11	II
**441**	Columbia-Rambouillet	Montana	1	5710	11	II
**442**	Columbia-Rambouillet	Montana	3	5660	11	II
**443**	Columbia-Rambouillet	Montana	2	5590	11	DD
**444**	Columbia-Rambouillet	Montana	4	5560	11	ID
**445**	Columbia-Rambouillet	Montana	3	5550	11	DD
**446**	Columbia-Rambouillet	Montana	3	5490	11	II
**447**	Columbia-Rambouillet	Montana	8	5460	11	DD
**448**	Columbia-Rambouillet	Montana	.	5340	11	DD
**449**	Columbia-Rambouillet	Montana	.	5340	11	DD
**450**	Columbia-Rambouillet	Montana	8	5340	11	ID
**451**	Columbia-Rambouillet	Montana	3	5270	11	DD
**452**	Columbia-Rambouillet	Montana	6	5260	11	DD
**453**	Columbia-Rambouillet	Montana	3	5230	11	DD
**454**	Columbia-Rambouillet	Montana	1	5170	11	ID
**455**	Columbia-Rambouillet	Montana	4	4950	11	II
**456**	Columbia-Rambouillet	Montana	2	4930	11	II
**457**	Columbia-Rambouillet	Montana	3	4910	11	II
**458**	Columbia-Rambouillet	Montana	4	4890	11	ID
**459**	Columbia-Rambouillet	Montana	6	4880	11	DD
**460**	Columbia-Rambouillet	Montana	2	4880	11	ID
**461**	Columbia-Rambouillet	Montana	2	4800	11	ID
**462**	Columbia-Rambouillet	Montana	3	4770	11	ID
**463**	Columbia-Rambouillet	Montana	6	4630	11	ID
**464**	Columbia-Rambouillet	Montana	.	4580	11	ID

**465**	Columbia-Rambouillet	Montana	4	4480	11	ID
**466**	Columbia-Rambouillet	Montana	2	4450	11	ID
**467**	Columbia-Rambouillet	Montana	1	4380	11	DD
**468**	Columbia-Rambouillet	Montana	2	4290	11	ID
**469**	Columbia-Rambouillet	Montana	2	4290	11	II
**470**	Columbia-Rambouillet	Montana	2	4240	11	DD
**471**	Columbia-Rambouillet	Montana	.	4220	11	ID
**472**	Columbia-Rambouillet	Montana	4	4200	11	ID
**473**	Columbia-Rambouillet	Montana	3	4200	11	ID
**474**	Columbia-Rambouillet	Montana	4	4170	11	DD
**475**	Columbia-Rambouillet	Montana	5	4160	11	ID
**476**	Columbia-Rambouillet	Montana	2	4120	11	DD
**477**	Columbia-Rambouillet	Montana	2	4120	11	ID
**478**	Columbia-Rambouillet	Montana	3	3990	11	ID
**479**	Columbia-Rambouillet	Montana	3	3970	11	DD
**480**	Columbia-Rambouillet	Montana	2	3940	11	ID
**481**	Columbia-Rambouillet	Montana	4	3910	11	ID
**482**	Columbia-Rambouillet	Montana	4	3880	11	II
**483**	Columbia-Rambouillet	Montana	3	3830	11	ID
**484**	Columbia-Rambouillet	Montana	3	3790	11	II
**485**	Columbia-Rambouillet	Montana	1	3790	11	ID
**486**	Columbia-Rambouillet	Montana	2	3780	11	ID
**487**	Columbia-Rambouillet	Montana	2	3780	11	ID
**488**	Columbia-Rambouillet	Montana	8	3760	11	ID
**489**	Columbia-Rambouillet	Montana	3	3760	11	II
**490**	Columbia-Rambouillet	Montana	4	3670	11	DD
**491**	Columbia-Rambouillet	Montana	5	3590	11	II
**492**	Columbia-Rambouillet	Montana	6	3570	11	ID
**493**	Columbia-Rambouillet	Montana	4	3550	11	II
**494**	Columbia-Rambouillet	Montana	3	3500	11	II
**495**	Columbia-Rambouillet	Montana	3	3450	11	ID
**496**	Columbia-Rambouillet	Montana	.	3410	11	ID
**497**	Columbia-Rambouillet	Montana	4	3310	11	II
**498**	Columbia-Rambouillet	Montana	3	3270	11	ID
**499**	Columbia-Rambouillet	Montana	4	3240	11	ID
**500**	Columbia-Rambouillet	Montana	3	3190	11	ID
**501**	Columbia-Rambouillet	Montana	3	3170	11	ID
**502**	Columbia-Rambouillet	Montana	4	3170	11	II
**503**	Columbia-Rambouillet	Montana	1	3150	11	DD
**504**	Columbia-Rambouillet	Montana	2	3130	11	DD
**505**	Columbia-Rambouillet	Montana	3	3090	11	DD
**506**	Columbia-Rambouillet	Montana	1	3070	11	ID
**507**	Columbia-Rambouillet	Montana	.	3060	11	II

**508**	Columbia-Rambouillet	Montana	3	3000	11	ID
**509**	Columbia-Rambouillet	Montana	3	3000	11	ID
**510**	Columbia-Rambouillet	Montana	.	2890	11	DD
**511**	Columbia-Rambouillet	Montana	6	2870	11	II
**512**	Columbia-Rambouillet	Montana	.	2870	11	ID
**513**	Columbia-Rambouillet	Montana	2	2850	11	ID
**514**	Columbia-Rambouillet	Montana	3	2820	11	ID
**515**	Columbia-Rambouillet	Montana	6	2820	11	DD
**516**	Columbia-Rambouillet	Montana	1	2800	11	ID
**517**	Columbia-Rambouillet	Montana	2	2790	11	DD
**518**	Columbia-Rambouillet	Montana	.	2750	11	ID
**519**	Columbia-Rambouillet	Montana	5	2720	11	II
**520**	Columbia-Rambouillet	Montana	6	2720	11	DD
**521**	Columbia-Rambouillet	Montana	7	2700	11	II
**522**	Columbia-Rambouillet	Montana	3	2670	11	DD
**523**	Columbia-Rambouillet	Montana	3	2660	11	ID
**524**	Columbia-Rambouillet	Montana	4	2650	11	II
**525**	Columbia-Rambouillet	Montana	3	2620	11	DD
**526**	Columbia-Rambouillet	Montana	5	2610	11	II
**527**	Columbia-Rambouillet	Montana	2	2540	11	II
**528**	Columbia-Rambouillet	Montana	3	2530	11	ID
**529**	Columbia-Rambouillet	Montana	4	2490	11	II
**530**	Columbia-Rambouillet	Montana	7	2470	11	II
**531**	Columbia-Rambouillet	Montana	5	2440	11	II
**532**	Columbia-Rambouillet	Montana	2	2420	11	ID
**533**	Columbia-Rambouillet	Montana	4	2420	11	DD
**534**	Columbia-Rambouillet	Montana	4	2410	11	DD
**535**	Columbia-Rambouillet	Montana	3	2410	11	ID
**536**	Columbia-Rambouillet	Montana	.	2410	11	ID
**537**	Columbia-Rambouillet	Montana	3	2410	11	II
**538**	Columbia-Rambouillet	Montana	6	2390	11	ID
**539**	Columbia-Rambouillet	Montana	6	2380	11	ID
**540**	Columbia-Rambouillet	Montana	1	2210	11	II
**541**	Columbia-Rambouillet	Montana	3	2180	11	II
**542**	Columbia-Rambouillet	Montana	7	2180	11	II
**543**	Columbia-Rambouillet	Montana	1	2170	11	ID
**544**	Columbia-Rambouillet	Montana	1	2160	11	II
**545**	Columbia-Rambouillet	Montana	3	2160	11	ID
**546**	Columbia-Rambouillet	Montana	2	2150	11	ID
**547**	Columbia-Rambouillet	Montana	8	2120	11	ID
**548**	Columbia-Rambouillet	Montana	3	2100	11	II
**549**	Columbia-Rambouillet	Montana	6	2080	11	II
**550**	Columbia-Rambouillet	Montana	4	2050	11	II

**551**	Columbia-Rambouillet	Montana	6	2050	11	ID
**552**	Columbia-Rambouillet	Montana	4	2030	11	II
**553**	Columbia-Rambouillet	Montana	1	2030	11	ID
**554**	Columbia-Rambouillet	Montana	7	2010	11	ID
**555**	Columbia-Rambouillet	Montana	4	1980	11	DD
**556**	Columbia-Rambouillet	Montana	1	1960	11	ID
**557**	Columbia-Rambouillet	Montana	1	1960	11	DD
**558**	Columbia-Rambouillet	Montana	7	1950	11	II
**559**	Columbia-Rambouillet	Montana	3	1930	11	II
**560**	Columbia-Rambouillet	Montana	2	1920	11	ID
**561**	Columbia-Rambouillet	Montana	2	1920	11	DD
**562**	Columbia-Rambouillet	Montana	4	1920	11	II
**563**	Columbia-Rambouillet	Montana	2	1910	11	DD
**564**	Columbia-Rambouillet	Montana	3	1900	11	II
**565**	Columbia-Rambouillet	Montana	.	1890	11	DD
**566**	Columbia-Rambouillet	Montana	.	1870	11	ID
**567**	Columbia-Rambouillet	Montana	3	1860	11	ID
**568**	Columbia-Rambouillet	Montana	2	1860	11	ID
**569**	Columbia-Rambouillet	Montana	4	1840	11	ID
**570**	Columbia-Rambouillet	Montana	2	1840	11	DD
**571**	Columbia-Rambouillet	Montana	2	1810	11	ID
**572**	Columbia-Rambouillet	Montana	7	1800	11	ID
**573**	Columbia-Rambouillet	Montana	.	1800	11	ID
**574**	Columbia-Rambouillet	Montana	3	1790	11	DD
**575**	Columbia-Rambouillet	Montana	.	1770	11	II
**576**	Columbia-Rambouillet	Montana	2	1760	11	II
**577**	Columbia-Rambouillet	Montana	6	1760	11	ID
**578**	Columbia-Rambouillet	Montana	6	1760	11	DD
**579**	Columbia-Rambouillet	Montana	2	1740	11	ID
**580**	Columbia-Rambouillet	Montana	1	1740	11	DD
**581**	Columbia-Rambouillet	Montana	.	1730	11	.
**582**	Columbia-Rambouillet	Montana	4	1720	11	ID
**583**	Columbia-Rambouillet	Montana	3	1650	11	ID
**584**	Columbia-Rambouillet	Montana	3	1630	11	II
**585**	Columbia-Rambouillet	Montana	2	1620	11	ID
**586**	Columbia-Rambouillet	Montana	3	1600	11	ID
**587**	Columbia-Rambouillet	Montana	3	1580	11	II
**588**	Columbia-Rambouillet	Montana	2	1540	11	ID
**589**	Columbia-Rambouillet	Montana	7	1530	11	ID
**590**	Columbia-Rambouillet	Montana	3	1530	11	ID
**591**	Columbia-Rambouillet	Montana	4	1500	11	II
**592**	Columbia-Rambouillet	Montana	2	1490	11	ID
**593**	Columbia-Rambouillet	Montana	8	1490	11	II

**594**	Columbia-Rambouillet	Montana	1	1460	11	II
**595**	Columbia-Rambouillet	Montana	6	1450	11	II
**596**	Columbia-Rambouillet	Montana	2	1440	11	ID
**597**	Columbia-Rambouillet	Montana	.	1440	11	DD
**598**	Columbia-Rambouillet	Montana	1	1420	11	II
**599**	Columbia-Rambouillet	Montana	4	1400	11	ID
**600**	Columbia-Rambouillet	Montana	1	1390	11	II
**601**	Columbia-Rambouillet	Montana	.	1380	11	II
**602**	Columbia-Rambouillet	Montana	3	1380	11	II
**603**	Columbia-Rambouillet	Montana	8	1370	11	ID
**604**	Columbia-Rambouillet	Montana	3	1370	11	ID
**605**	Columbia-Rambouillet	Montana	.	1350	11	ID
**606**	Columbia-Rambouillet	Montana	4	1340	11	II
**607**	Columbia-Rambouillet	Montana	4	1340	11	DD
**608**	Columbia-Rambouillet	Montana	5	1330	11	DD
**609**	Columbia-Rambouillet	Montana	1	1310	11	DD
**610**	Columbia-Rambouillet	Montana	2	1290	11	DD
**611**	Columbia-Rambouillet	Montana	3	1260	11	DD
**612**	Columbia-Rambouillet	Montana	4	1240	11	ID
**613**	Columbia-Rambouillet	Montana	1	1240	11	II
**614**	Columbia-Rambouillet	Montana	1	1220	11	ID
**615**	Columbia-Rambouillet	Montana	2	1220	11	II
**616**	Columbia-Rambouillet	Montana	1	1210	11	ID
**617**	Columbia-Rambouillet	Montana	1	1190	11	DD
**618**	Columbia-Rambouillet	Montana	2	1190	11	DD
**619**	Columbia-Rambouillet	Montana	2	1180	11	ID
**620**	Columbia-Rambouillet	Montana	7	1180	11	DD
**621**	Columbia-Rambouillet	Montana	1	1170	11	II
**622**	Columbia-Rambouillet	Montana	3	1160	11	ID
**623**	Columbia-Rambouillet	Montana	1	1140	11	ID
**624**	Columbia-Rambouillet	Montana	4	1140	11	DD
**625**	Columbia-Rambouillet	Montana	.	1130	11	ID
**626**	Columbia-Rambouillet	Montana	4	1130	11	II
**627**	Columbia-Rambouillet	Montana	4	1120	11	II
**628**	Columbia-Rambouillet	Montana	3	1120	11	ID
**629**	Columbia-Rambouillet	Montana	2	1090	11	ID
**630**	Columbia-Rambouillet	Montana	1	1090	11	ID
**631**	Columbia-Rambouillet	Montana	2	1090	11	DD
**632**	Columbia-Rambouillet	Montana	7	1080	11	II
**633**	Columbia-Rambouillet	Montana	2	1070	11	DD
**634**	Columbia-Rambouillet	Montana	2	1060	11	ID
**635**	Columbia-Rambouillet	Montana	8	1050	11	ID
**636**	Columbia-Rambouillet	Montana	.	1050	11	II

**637**	Columbia-Rambouillet	Montana	1	1040	11	II
**638**	Columbia-Rambouillet	Montana	1	1040	11	II
**639**	Columbia-Rambouillet	Montana	3	1030	11	II
**640**	Columbia-Rambouillet	Montana	3	1020	11	II
**641**	Columbia-Rambouillet	Montana	1	1010	11	ID
**642**	Columbia-Rambouillet	Montana	3	976	11	ID
**643**	Columbia-Rambouillet	Montana	8	965	11	II
**644**	Columbia-Rambouillet	Montana	1	957	11	ID
**645**	Columbia-Rambouillet	Montana	.	947	11	ID
**646**	Columbia-Rambouillet	Montana	1	939	11	ID
**647**	Columbia-Rambouillet	Montana	7	937	11	DD
**648**	Columbia-Rambouillet	Montana	.	930	11	II
**649**	Columbia-Rambouillet	Montana	2	930	11	ID
**650**	Columbia-Rambouillet	Montana	.	888	11	II
**651**	Columbia-Rambouillet	Montana	2	875	11	ID
**652**	Columbia-Rambouillet	Montana	4	874	11	II
**653**	Columbia-Rambouillet	Montana	2	863	11	II
**654**	Columbia-Rambouillet	Montana	.	859	11	DD
**655**	Columbia-Rambouillet	Montana	1	855	11	ID
**656**	Columbia-Rambouillet	Montana	3	850	11	ID
**657**	Columbia-Rambouillet	Montana	2	843	11	II
**658**	Columbia-Rambouillet	Montana	1	810	11	II
**659**	Columbia-Rambouillet	Montana	.	808	11	DD
**660**	Columbia-Rambouillet	Montana	2	801	11	ID
**661**	Columbia-Rambouillet	Montana	3	800	11	II
**662**	Columbia-Rambouillet	Montana	6	787	11	ID
**663**	Columbia-Rambouillet	Montana	3	777	11	ID
**664**	Columbia-Rambouillet	Montana	2	773	11	II
**665**	Columbia-Rambouillet	Montana	.	761	11	ID
**666**	Columbia-Rambouillet	Montana	6	760	11	ID
**667**	Columbia-Rambouillet	Montana	3	756	11	II
**668**	Columbia-Rambouillet	Montana	2	756	11	ID
**669**	Columbia-Rambouillet	Montana	6	749	11	II
**670**	Columbia-Rambouillet	Montana	4	749	11	II
**671**	Columbia-Rambouillet	Montana	4	739	11	ID
**672**	Columbia-Rambouillet	Montana	4	739	11	ID
**673**	Columbia-Rambouillet	Montana	2	732	11	ID
**674**	Columbia-Rambouillet	Montana	2	704	11	ID
**675**	Columbia-Rambouillet	Montana	1	694	11	ID
**676**	Columbia-Rambouillet	Montana	3	694	11	ID
**677**	Columbia-Rambouillet	Montana	3	694	11	II
**678**	Columbia-Rambouillet	Montana	6	678	11	DD
**679**	Columbia-Rambouillet	Montana	1	674	11	DD

**680**	Columbia-Rambouillet	Montana	6	671	11	ID
**681**	Columbia-Rambouillet	Montana	4	668	11	II
**682**	Columbia-Rambouillet	Montana	1	639	11	ID
**683**	Columbia-Rambouillet	Montana	4	629	11	ID
**684**	Columbia-Rambouillet	Montana	2	606	11	ID
**685**	Columbia-Rambouillet	Montana	3	606	11	ID
**686**	Columbia-Rambouillet	Montana	4	606	11	ID
**687**	Columbia-Rambouillet	Montana	2	595	11	II
**688**	Columbia-Rambouillet	Montana	1	593	11	DD
**689**	Columbia-Rambouillet	Montana	4	586	11	DD
**690**	Columbia-Rambouillet	Montana	.	583	11	DD
**691**	Columbia-Rambouillet	Montana	.	582	11	ID
**692**	Columbia-Rambouillet	Montana	3	580	11	ID
**693**	Columbia-Rambouillet	Montana	3	575	11	ID
**694**	Columbia-Rambouillet	Montana	2	565	11	ID
**695**	Columbia-Rambouillet	Montana	2	565	11	DD
**696**	Columbia-Rambouillet	Montana	1	564	11	II
**697**	Columbia-Rambouillet	Montana	1	555	11	ID
**698**	Columbia-Rambouillet	Montana	6	554	11	II
**699**	Columbia-Rambouillet	Montana	1	545	11	DD
**700**	Columbia-Rambouillet	Montana	4	536	11	ID
**701**	Columbia-Rambouillet	Montana	3	531	11	ID
**702**	Columbia-Rambouillet	Montana	.	531	11	II
**703**	Columbia-Rambouillet	Montana	1	529	11	ID
**704**	Columbia-Rambouillet	Montana	5	521	11	ID
**705**	Columbia-Rambouillet	Montana	2	520	11	ID
**706**	Columbia-Rambouillet	Montana	.	518	11	II
**707**	Columbia-Rambouillet	Montana	6	506	11	II
**708**	Columbia-Rambouillet	Montana	1	503	11	ID
**709**	Columbia-Rambouillet	Montana	2	493	11	DD
**710**	Columbia-Rambouillet	Montana	3	481	11	II
**711**	Columbia-Rambouillet	Montana	2	472	11	ID
**712**	Columbia-Rambouillet	Montana	2	468	11	DD
**713**	Columbia-Rambouillet	Montana	4	467	11	II
**714**	Columbia-Rambouillet	Montana	3	464	11	ID
**715**	Columbia-Rambouillet	Montana	1	461	11	II
**716**	Columbia-Rambouillet	Montana	6	459	11	DD
**717**	Columbia-Rambouillet	Montana	1	445	11	ID
**718**	Columbia-Rambouillet	Montana	.	445	11	ID
**719**	Columbia-Rambouillet	Montana	1	442	11	II
**720**	Columbia-Rambouillet	Montana	.	433	11	ID
**721**	Columbia-Rambouillet	Montana	2	430	11	II
**722**	Columbia-Rambouillet	Montana	1	420	11	ID

**723**	Columbia-Rambouillet	Montana	3	406	11	ID
**724**	Columbia-Rambouillet	Montana	2	402	11	ID
**725**	Columbia-Rambouillet	Montana	8	397	11	ID
**726**	Columbia-Rambouillet	Montana	1	395	11	DD
**727**	Columbia-Rambouillet	Montana	2	395	11	DD
**728**	Columbia-Rambouillet	Montana	.	392	11	ID
**729**	Columbia-Rambouillet	Montana	3	392	11	ID
**730**	Columbia-Rambouillet	Montana	2	387	11	ID
**731**	Columbia-Rambouillet	Montana	3	381	11	DD
**732**	Columbia-Rambouillet	Montana	4	381	11	DD
**733**	Columbia-Rambouillet	Montana	1	365	11	ID
**734**	Columbia-Rambouillet	Montana	1	365	11	ID
**735**	Columbia-Rambouillet	Montana	3	364	11	II
**736**	Columbia-Rambouillet	Montana	2	363	11	ID
**737**	Columbia-Rambouillet	Montana	1	354	11	ID
**738**	Columbia-Rambouillet	Montana	1	351	11	II
**739**	Columbia-Rambouillet	Montana	3	347	11	ID
**740**	Columbia-Rambouillet	Montana	1	342	11	ID
**741**	Columbia-Rambouillet	Montana	1	339	11	II
**742**	Columbia-Rambouillet	Montana	6	334	11	ID
**743**	Columbia-Rambouillet	Montana	5	333	11	II
**744**	Columbia-Rambouillet	Montana	2	329	11	DD
**745**	Columbia-Rambouillet	Montana	3	329	11	II
**746**	Columbia-Rambouillet	Montana	3	326	11	ID
**747**	Columbia-Rambouillet	Montana	4	319	11	ID
**748**	Columbia-Rambouillet	Montana	5	318	11	.
**749**	Columbia-Rambouillet	Montana	.	314	11	DD
**750**	Columbia-Rambouillet	Montana	2	310	11	II
**751**	Columbia-Rambouillet	Montana	2	305	11	ID
**752**	Columbia-Rambouillet	Montana	3	303	11	II
**753**	Columbia-Rambouillet	Montana	1	298	11	ID
**754**	Columbia-Rambouillet	Montana	1	297	11	II
**755**	Columbia-Rambouillet	Montana	1	296	11	ID
**756**	Columbia-Rambouillet	Montana	2	287	11	ID
**757**	Columbia-Rambouillet	Montana	4	280	11	ID
**758**	Columbia-Rambouillet	Montana	6	274	11	DD
**759**	Columbia-Rambouillet	Montana	1	273	11	ID
**760**	Columbia-Rambouillet	Montana	2	265	11	DD
**761**	Columbia-Rambouillet	Montana	4	265	11	II
**762**	Columbia-Rambouillet	Montana	2	263	11	DD
**763**	Columbia-Rambouillet	Montana	5	253	11	ID
**764**	Columbia-Rambouillet	Montana	3	248	11	ID
**765**	Columbia-Rambouillet	Montana	2	241	11	ID

**766**	Columbia-Rambouillet	Montana	8	235	11	II
**767**	Columbia-Rambouillet	Montana	4	231	11	ID
**768**	Columbia-Rambouillet	Montana	1	221	11	II
**769**	Columbia-Rambouillet	Montana	2	217	11	DD
**770**	Columbia-Rambouillet	Montana	2	215	11	II
**771**	Columbia-Rambouillet	Montana	2	214	11	ID
**772**	Columbia-Rambouillet	Montana	2	210	11	II
**773**	Columbia-Rambouillet	Montana	1	206	11	DD
**774**	Columbia-Rambouillet	Montana	2	201	11	DD
**775**	Columbia-Rambouillet	Montana	2	198	11	II
**776**	Columbia-Rambouillet	Montana	4	196	11	ID
**777**	Columbia-Rambouillet	Montana	1	195	11	II
**778**	Columbia-Rambouillet	Montana	8	193	11	ID
**779**	Columbia-Rambouillet	Montana	2	191	11	DD
**780**	Columbia-Rambouillet	Montana	2	190	11	ID
**781**	Columbia-Rambouillet	Montana	1	188	11	ID
**782**	Columbia-Rambouillet	Montana	6	187	11	DD
**783**	Columbia-Rambouillet	Montana	2	182	11	ID
**784**	Columbia-Rambouillet	Montana	2	181	11	DD
**785**	Columbia-Rambouillet	Montana	5	175	11	DD
**786**	Columbia-Rambouillet	Montana	4	175	11	ID
**787**	Columbia-Rambouillet	Montana	.	175	11	DD
**788**	Columbia-Rambouillet	Montana	1	174	11	DD
**789**	Columbia-Rambouillet	Montana	2	173	11	DD
**790**	Columbia-Rambouillet	Montana	4	172	11	DD
**791**	Columbia-Rambouillet	Montana	8	171	11	ID
**792**	Columbia-Rambouillet	Montana	2	167	11	II
**793**	Columbia-Rambouillet	Montana	2	165	11	ID
**794**	Columbia-Rambouillet	Montana	4	165	11	II
**795**	Columbia-Rambouillet	Montana	1	164	11	ID
**796**	Columbia-Rambouillet	Montana	1	163	11	II
**797**	Columbia-Rambouillet	Montana	1	162	11	II
**798**	Columbia-Rambouillet	Montana	7	161	11	ID
**799**	Columbia-Rambouillet	Montana	.	155	11	II
**800**	Columbia-Rambouillet	Montana	2	152	11	DD
**801**	Columbia-Rambouillet	Montana	3	151	11	II
**802**	Columbia-Rambouillet	Montana	.	144	11	ID
**803**	Columbia-Rambouillet	Montana	1	143	11	DD
**804**	Columbia-Rambouillet	Montana	3	142	11	DD
**805**	Columbia-Rambouillet	Montana	1	142	11	DD
**806**	Columbia-Rambouillet	Montana	2	141	11	II
**807**	Columbia-Rambouillet	Montana	1	140	11	ID
**808**	Columbia-Rambouillet	Montana	.	139	11	II

**809**	Columbia-Rambouillet	Montana	3	139	11	ID
**810**	Columbia-Rambouillet	Montana	8	138	11	DD
**811**	Columbia-Rambouillet	Montana	2	138	11	II
**812**	Columbia-Rambouillet	Montana	1	136	11	ID
**813**	Columbia-Rambouillet	Montana	3	135	11	ID
**814**	Columbia-Rambouillet	Montana	2	133	11	ID
**815**	Columbia-Rambouillet	Montana	3	132	11	II
**816**	Columbia-Rambouillet	Montana	.	128	11	II
**817**	Columbia-Rambouillet	Montana	.	128	11	DD
**818**	Columbia-Rambouillet	Montana	1	125	11	II
**819**	Columbia-Rambouillet	Montana	3	124	11	II
**820**	Columbia-Rambouillet	Montana	1	118	11	DD
**821**	Columbia-Rambouillet	Montana	1	117	11	ID
**822**	Columbia-Rambouillet	Montana	1	117	11	ID
**823**	Columbia-Rambouillet	Montana	2	117	11	II
**824**	Columbia-Rambouillet	Montana	1	116	11	ID
**825**	Columbia-Rambouillet	Montana	7	113	11	II
**826**	Columbia-Rambouillet	Montana	2	112	11	DD
**827**	Columbia-Rambouillet	Montana	3	112	11	ID
**828**	Columbia-Rambouillet	Montana	4	108	11	II
**829**	Columbia-Rambouillet	Montana	2	107	11	ID
**830**	Columbia-Rambouillet	Montana	4	99.8	11	II
**831**	Columbia-Rambouillet	Montana	4	98.3	11	DD
**832**	Columbia-Rambouillet	Montana	7	97.8	11	II
**833**	Columbia-Rambouillet	Montana	3	93.5	11	ID
**834**	Columbia-Rambouillet	Montana	3	93.4	11	II
**835**	Columbia-Rambouillet	Montana	1	91.6	11	ID
**836**	Columbia-Rambouillet	Montana	1	91.4	11	ID
**837**	Columbia-Rambouillet	Montana	2	84.6	11	DD
**838**	Columbia-Rambouillet	Montana	1	84.1	11	II
**839**	Columbia-Rambouillet	Montana	3	81.5	11	II
**840**	Columbia-Rambouillet	Montana	2	80.9	11	II
**841**	Columbia-Rambouillet	Montana	4	80.9	11	II
**842**	Columbia-Rambouillet	Montana	1	79.8	11	II
**843**	Columbia-Rambouillet	Montana	1	78.4	11	ID
**844**	Columbia-Rambouillet	Montana	3	77.3	11	ID
**845**	Columbia-Rambouillet	Montana	1	71.5	11	DD
**846**	Columbia-Rambouillet	Montana	.	70.7	11	ID
**847**	Columbia-Rambouillet	Montana	3	70.1	11	II
**848**	Columbia-Rambouillet	Montana	2	68.5	11	ID
**849**	Columbia-Rambouillet	Montana	1	68.2	11	ID
**850**	Columbia-Rambouillet	Montana	1	68	11	DD
**851**	Columbia-Rambouillet	Montana	1	66.6	11	DD

**852**	Columbia-Rambouillet	Montana	1	66.6	11	II
**853**	Columbia-Rambouillet	Montana	6	66.5	11	DD
**854**	Columbia-Rambouillet	Montana	3	64.6	11	DD
**855**	Columbia-Rambouillet	Montana	3	57.8	11	DD
**856**	Columbia-Rambouillet	Montana	3	56.4	11	ID
**857**	Columbia-Rambouillet	Montana	2	55.3	11	DD
**858**	Columbia-Rambouillet	Montana	3	55.2	11	ID
**859**	Columbia-Rambouillet	Montana	4	53.9	11	ID
**860**	Columbia-Rambouillet	Montana	1	53.5	11	ID
**861**	Columbia-Rambouillet	Montana	.	52.2	11	DD
**862**	Columbia-Rambouillet	Montana	2	50.5	11	II
**863**	Columbia-Rambouillet	Montana	2	50.4	11	II
**864**	Columbia-Rambouillet	Montana	3	48.4	11	ID
**865**	Columbia-Rambouillet	Montana	3	48.2	11	II
**866**	Columbia-Rambouillet	Montana	1	47.3	11	ID
**867**	Columbia-Rambouillet	Montana	2	45	11	II
**868**	Columbia-Rambouillet	Montana	2	41.6	11	II
**869**	Columbia-Rambouillet	Montana	.	40.7	11	DD
**870**	Columbia-Rambouillet	Montana	1	40.6	11	DD
**871**	Columbia-Rambouillet	Montana	3	40.4	11	DD
**872**	Columbia-Rambouillet	Montana	3	40	11	ID
**873**	Columbia-Rambouillet	Montana	5	39.2	11	ID
**874**	Columbia-Rambouillet	Montana	8	39	11	II
**875**	Columbia-Rambouillet	Montana	6	39	11	DD
**876**	Columbia-Rambouillet	Montana	.	37.4	11	.
**877**	Columbia-Rambouillet	Montana	.	35.5	11	.
**878**	Columbia-Rambouillet	Montana	5	33.8	11	ID
**879**	Columbia-Rambouillet	Montana	3	32.9	11	ID
**880**	Columbia-Rambouillet	Montana	1	31.8	11	II
**881**	Columbia-Rambouillet	Montana	1	28.5	11	ID
**882**	Columbia-Rambouillet	Montana	.	27.9	11	DD
**883**	Columbia-Rambouillet	Montana	1	27.2	11	ID
**884**	Columbia-Rambouillet	Montana	3	26	11	II
**885**	Columbia-Rambouillet	Montana	1	24.6	11	ID
**886**	Columbia-Rambouillet	Montana	3	24.3	11	DD
**887**	Columbia-Rambouillet	Montana	3	24.1	11	ID
**888**	Columbia-Rambouillet	Montana	6	22.2	11	ID
**889**	Columbia-Rambouillet	Montana	.	22	11	II
**890**	Columbia-Rambouillet	Montana	7	21.8	11	DD
**891**	Columbia-Rambouillet	Montana	2	21.3	11	ID
**892**	Columbia-Rambouillet	Montana	.	20.8	11	ID
**893**	Columbia-Rambouillet	Montana	3	20.3	11	ID
**894**	Columbia-Rambouillet	Montana	.	20.1	11	II

**895**	Columbia-Rambouillet	Montana	3	19.7	11	II
**896**	Columbia-Rambouillet	Montana	1	19.6	11	DD
**897**	Columbia-Rambouillet	Montana	1	19.1	11	DD
**898**	Columbia-Rambouillet	Montana	1	19	11	ID
**899**	Columbia-Rambouillet	Montana	1	17.3	11	ID
**900**	Columbia-Rambouillet	Montana	.	17.1	11	II
**901**	Columbia-Rambouillet	Montana	2	16	11	ID
**902**	Columbia-Rambouillet	Montana	1	14.6	11	II
**903**	Columbia-Rambouillet	Montana	3	14.5	11	ID
**904**	Columbia-Rambouillet	Montana	1	13.3	11	ID
**905**	Columbia-Rambouillet	Montana	3	13	11	II
**906**	Columbia-Rambouillet	Montana	3	12.2	11	II
**907**	Columbia-Rambouillet	Montana	1	11.8	11	ID
**908**	Columbia-Rambouillet	Montana	.	11.4	11	ID
**909**	Columbia-Rambouillet	Montana	3	11.2	11	II
**910**	Columbia-Rambouillet	Montana	3	11.1	11	ID
**911**	Columbia-Rambouillet	Montana	1	11	11	II
**912**	Columbia-Rambouillet	Montana	3	.	11	ID
**913**	Columbia-Rambouillet	Montana	5	.	11	DD
**914**	Columbia-Rambouillet	Montana	3	.	11	ID
**915**	Columbia-Rambouillet	Montana	7	.	11	DD
**916**	Columbia-Rambouillet	Montana	3	.	11	DD
**917**	Columbia-Rambouillet	Montana	3	.	11	DD
**918**	Columbia-Rambouillet	Montana	6	.	11	DD
**919**	Columbia-Rambouillet	Montana	3	.	11	ID
**920**	Columbia-Rambouillet	Montana	3	.	11	ID
**921**	Columbia-Rambouillet	Montana	3	.	11	II
**922**	Columbia-Rambouillet	Montana	2	.	11	ID
**923**	Columbia-Rambouillet	Montana	5	.	11	ID
**924**	Columbia-Rambouillet	Montana	2	.	11	II
**925**	Columbia-Rambouillet	Montana	6	1830	14	DD
**926**	Columbia-Rambouillet	Montana	7	1250	14	II
**927**	Columbia-Rambouillet	Montana	.	823	14	DD
**928**	Columbia-Rambouillet	Montana	1	275	14	II
**929**	Columbia-Rambouillet	Montana	7	178	14	ID
**930**	Columbia-Rambouillet	Montana	1	172	14	ID
**931**	Columbia-Rambouillet	Montana	2	140	14	ID
**932**	Columbia-Rambouillet	Montana	7	51.6	14	II
**933**	Columbia-Rambouillet	Montana	.	55.8	23	ID
**934**	Columbia-Rambouillet	Montana	.	311	24	ID
**935**	Columbia-Rambouillet	Montana	.	173	24	II
**936**	Columbia-Rambouillet	Montana	.	27.3	24	II
**937**	Columbia	Idaho 2004	4	169	11	ID

**938**	Columbia	Idaho 2004	5	132	11	II
**939**	Columbia	Idaho 2004	4	992	11	ID
**940**	Columbia	Idaho 2004	5	.	11	ID
**941**	Columbia	Idaho 2004	6	21	11	II
**942**	Columbia	Idaho 2004	3	305	.	ID
**943**	Columbia	Idaho 2004	6	601	11	ID
**944**	Columbia	Idaho 2004	4	15	11	II
**945**	Columbia	Idaho 2004	4	.	11	ID
**946**	Columbia	Idaho 2004	4	7660	11	ID
**947**	Columbia	Idaho 2004	5	2	11	ID
**948**	Columbia	Idaho 2004	5	187	11	DD
**949**	Columbia	Idaho 2004	4	13400	11	DD
**950**	Columbia	Idaho 2004	4	104	.	DD
**951**	Columbia	Idaho 2004	6	1880	11	II
**952**	Columbia	Idaho 2004	3	.	11	ID
**953**	Columbia	Idaho 2004	6	63	11	II
**954**	Columbia	Idaho 2004	3	.	11	DD
**955**	Columbia	Idaho 2004	4	235	11	ID
**956**	Columbia	Idaho 2004	5	1130	11	II
**957**	Columbia	Idaho 2004	3	1360	11	ID
**958**	Columbia	Idaho 2004	6	63	.	DD
**959**	Columbia	Idaho 2004	5	1520	11	ID
**960**	Columbia	Idaho 2004	5	288	11	DD
**961**	Columbia	Idaho 2004	4	26	11	II
**962**	Columbia	Idaho 2004	6	11700	11	ID
**963**	Columbia	Idaho 2004	5	1170	11	ID
**964**	Columbia	Idaho 2004	6	755	11	ID
**965**	Columbia	Idaho 2004	4	613	11	DD
**966**	Columbia	Idaho 2004	5	34	.	ID
**967**	Columbia	Idaho 2004	3	.	11	ID
**968**	Columbia	Idaho 2004	4	183	11	II
**969**	Columbia	Idaho 2004	3	.	11	ID
**970**	Columbia	Idaho 2004	6	.	11	DD
**971**	Columbia	Idaho 2004	5	33	.	II
**972**	Columbia	Idaho 2004	6	218	.	ID
**973**	Columbia	Idaho 2004	4	241	11	ID
**974**	Columbia	Idaho 2004	3	34	11	DD
**975**	Columbia	Idaho 2004	3	.	11	II
**976**	Columbia	Idaho 2004	3	.	11	ID
**977**	Columbia	Idaho 2004	5	.	13	II
**978**	Columbia	Idaho 2004	3	233	11	ID
**979**	Columbia	Idaho 2004	3	321	11	ID
**980**	Columbia	Idaho 2004	5	267	.	II

**981**	Columbia	Idaho 2004	5	.	11	II
**982**	Columbia	Idaho 2004	6	267	.	DD
**983**	Columbia	Idaho 2004	3	.	11	ID
**984**	Columbia	Idaho 2004	3	.	11	ID
**985**	Columbia	Idaho 2004	5	7	11	DD
**986**	Columbia	Idaho 2004	3	721	11	ID
**987**	Columbia	Idaho 2004	3	.	11	ID
**988**	Columbia	Idaho 2004	5	2260	11	ID
**989**	Columbia	Idaho 2004	5	.	11	II
**990**	Columbia	Idaho 2004	5	2	11	DD
**991**	Columbia	Idaho 2004	3	.	.	ID
**992**	Columbia	Idaho 2004	5	98	11	DD
**993**	Columbia	Idaho 2004	3	.	11	ID
**994**	Columbia	Idaho 2004	3	305	11	ID
**995**	Columbia	Idaho 2004	4	416	.	ID
**996**	Columbia	Idaho 2004	3	.	11	II
**997**	Columbia	Idaho 2004	5	956	11	DD
**998**	Columbia	Idaho 2004	5	3910	11	ID
**999**	Columbia	Idaho 2004	6	605	11	ID
**1000**	Columbia	Idaho 2004	3	9	11	II
**1001**	Columbia	Idaho 2004	5	5240	11	II
**1002**	Columbia	Idaho 2004	5	416	11	ID
**1003**	Columbia	Idaho 2004	3	.	11	ID
**1004**	Columbia	Idaho 2004	3	470	11	DD
**1005**	Columbia	Idaho 2004	6	2590	11	ID
**1006**	Columbia	Idaho 2004	3	113	11	ID
**1007**	Columbia	Idaho 2004	4	3910	11	II
**1008**	Columbia	Idaho 2004	5	.	11	ID
**1009**	Columbia	Idaho 2004	4	31	.	DD
**1010**	Columbia	Idaho 2004	6	9	11	ID
**1011**	Columbia	Idaho 2004	6	576	11	ID
**1012**	Columbia	Idaho 2004	6	58	11	ID
**1013**	Columbia	Idaho 2004	3	.	.	ID
**1014**	Columbia	Idaho 2004	6	2550	11	ID
**1015**	Columbia	Idaho 2004	3	441	.	ID
**1016**	Columbia	Idaho 2004	3	.	11	ID
**1017**	Columbia	Idaho 2004	4	.	11	ID
**1018**	Columbia	Idaho 2004	3	213	11	II
**1019**	Columbia	Idaho 2004	3	.	11	ID
**1020**	Columbia	Idaho 2004	6	1540	11	II
**1021**	Columbia	Idaho 2004	3	403	11	ID
**1022**	Columbia	Idaho 2004	3	50600	11	ID
**1023**	Columbia	Idaho 2004	3	297	11	DD

**1024**	Columbia	Idaho 2004	4	.	11	ID
**1025**	Columbia	Idaho 2004	6	921	.	ID
**1026**	Columbia	Idaho 2004	5	531	11	II
**1027**	Columbia	Idaho 2004	6	193	11	ID
**1028**	Columbia	Idaho 2004	3	.	11	ID
**1029**	Columbia	Idaho 2004	6	928	11	II
**1030**	Columbia	Idaho 2004	3	693	11	II
**1031**	Columbia	Idaho 2004	5	824	11	ID
**1032**	Columbia	Idaho 2004	3	.	11	II
**1033**	Columbia	Idaho 2004	5	4130	11	ID
**1034**	Columbia	Idaho 2004	3	702	11	ID
**1035**	Columbia	Idaho 2004	4	.	11	DD
**1036**	Columbia	Idaho 2004	4	1880	11	ID
**1037**	Columbia	Idaho 2004	4	.	11	ID
**1038**	Columbia	Idaho 2004	4	411	11	ID
**1039**	Columbia	Idaho 2004	4	.	11	ID
**1040**	Columbia	Idaho 2004	3	.	11	II
**1041**	Columbia	Idaho 2004	4	.	.	II
**1042**	Columbia	Idaho 2004	5	448	11	II
**1043**	Columbia	Idaho 2004	4	892	11	II
**1044**	Columbia	Idaho 2004	5	1460	11	ID
**1045**	Polypay	Idaho 2004	3	.	.	II
**1046**	Polypay	Idaho 2004	5	.	13	II
**1047**	Polypay	Idaho 2004	5	2290	11	ID
**1048**	Polypay	Idaho 2004	5.5	19000	12	II
**1049**	Polypay	Idaho 2004	5.5	6230	11	II
**1050**	Polypay	Idaho 2004	6	.	11	II
**1051**	Polypay	Idaho 2004	5	5640	12	ID
**1052**	Polypay	Idaho 2004	3	.	11	II
**1053**	Polypay	Idaho 2004	6	303	11	ID
**1054**	Polypay	Idaho 2004	6	118	11	ID
**1055**	Polypay	Idaho 2004	5	234	11	ID
**1056**	Polypay	Idaho 2004	5	.	11	II
**1057**	Polypay	Idaho 2004	4	.	11	ID
**1058**	Polypay	Idaho 2004	6	12300	.	ID
**1059**	Polypay	Idaho 2004	5	636	11	ID
**1060**	Polypay	Idaho 2004	5	.	11	ID
**1061**	Polypay	Idaho 2004	4	339	11	ID
**1062**	Polypay	Idaho 2004	3	.	11	ID
**1063**	Polypay	Idaho 2004	3	72	11	ID
**1064**	Polypay	Idaho 2004	4	17	11	II
**1065**	Polypay	Idaho 2004	4	507	11	ID
**1066**	Polypay	Idaho 2004	5.5	150	11	ID

**1067**	Polypay	Idaho 2004	5	11900	11	II
**1068**	Polypay	Idaho 2004	5.5	.	11	II
**1069**	Polypay	Idaho 2004	3	.	11	ID
**1070**	Polypay	Idaho 2004	3	463	.	ID
**1071**	Polypay	Idaho 2004	3	558	11	ID
**1072**	Polypay	Idaho 2004	5.5	601	.	II
**1073**	Polypay	Idaho 2004	5	1590	.	II
**1074**	Polypay	Idaho 2004	5	852	13	II
**1075**	Polypay	Idaho 2004	4	683	13	ID
**1076**	Polypay	Idaho 2004	5	39	14	II
**1077**	Polypay	Idaho 2004	4	.	11	II
**1078**	Polypay	Idaho 2004	5.5	.	23	II
**1079**	Polypay	Idaho 2004	3	.	11	II
**1080**	Polypay	Idaho 2004	3	.	11	II
**1081**	Polypay	Idaho 2004	5	159	11	II
**1082**	Polypay	Idaho 2004	3	516	11	ID
**1083**	Polypay	Idaho 2004	6	72	13	II
**1084**	Polypay	Idaho 2004	5	2820	11	II
**1085**	Polypay	Idaho 2004	6	.	11	ID
**1086**	Polypay	Idaho 2004	3	961	12	II
**1087**	Polypay	Idaho 2004	3	1660	11	II
**1088**	Polypay	Idaho 2004	3	.	11	ID
**1089**	Polypay	Idaho 2004	3	143	11	II
**1090**	Polypay	Idaho 2004	4	.	11	II
**1091**	Polypay	Idaho 2004	4	.	11	ID
**1092**	Polypay	Idaho 2004	5	.	13	ID
**1093**	Polypay	Idaho 2004	5	264	11	II
**1094**	Polypay	Idaho 2004	3	.	11	II
**1095**	Polypay	Idaho 2004	3	.	.	II
**1096**	Polypay	Idaho 2004	6	33	11	DD
**1097**	Polypay	Idaho 2004	4	798	11	II
**1098**	Polypay	Idaho 2004	4	50	11	II
**1099**	Polypay	Idaho 2004	6	695	11	II
**1100**	Polypay	Idaho 2004	3	50	.	II
**1101**	Polypay	Idaho 2004	4	.	11	ID
**1102**	Polypay	Idaho 2004	4	408	.	II
**1103**	Polypay	Idaho 2004	4	62700	11	ID
**1104**	Polypay	Idaho 2004	4	.	11	II
**1105**	Polypay	Idaho 2004	5	.	11	II
**1106**	Polypay	Idaho 2004	3	.	14	ID
**1107**	Polypay	Idaho 2004	3	.	11	II
**1108**	Polypay	Idaho 2004	6	1170	11	ID
**1109**	Polypay	Idaho 2004	6	1150	11	ID

**1110**	Polypay	Idaho 2004	4	.	11	ID
**1111**	Polypay	Idaho 2004	3	.	11	II
**1112**	Polypay	Idaho 2004	4	.	11	ID
**1113**	Polypay	Idaho 2004	4	.	.	II
**1114**	Polypay	Idaho 2004	4	.	11	ID
**1115**	Polypay	Idaho 2004	4	.	11	II
**1116**	Polypay	Idaho 2004	6	.	12	ID
**1117**	Polypay	Idaho 2004	5	.	11	ID
**1118**	Polypay	Idaho 2004	5	59	.	ID
**1119**	Polypay	Idaho 2004	3	46	11	II
**1120**	Polypay	Idaho 2004	3	.	11	II
**1121**	Polypay	Idaho 2004	4	1120	11	II
**1122**	Polypay	Idaho 2004	5	.	11	II
**1123**	Polypay	Idaho 2004	3	1080	11	II
**1124**	Polypay	Idaho 2004	3	221	13	ID
**1125**	Polypay	Idaho 2004	5	1420	11	ID
**1126**	Polypay	Idaho 2004	6	.	11	ID
**1127**	Polypay	Idaho 2004	5	0	11	II
**1128**	Polypay	Idaho 2004	4	.	11	II
**1129**	Polypay	Idaho 2004	4	.	11	DD
**1130**	Polypay	Idaho 2004	6	332	.	II
**1131**	Polypay	Idaho 2004	4	.	11	II
**1132**	Polypay	Idaho 2004	5	297	11	II
**1133**	Polypay	Idaho 2004	6	1420	11	II
**1134**	Polypay	Idaho 2004	6	360	11	II
**1135**	Polypay	Idaho 2004	4	84	.	II
**1136**	Polypay	Idaho 2004	4	6510	.	ID
**1137**	Polypay	Idaho 2004	3	.	11	ID
**1138**	Polypay	Idaho 2004	4	.	11	ID
**1139**	Polypay	Idaho 2004	3	199	11	ID
**1140**	Polypay	Idaho 2004	3	.	12	II
**1141**	Polypay	Idaho 2004	5	3870	11	II
**1142**	Polypay	Idaho 2004	5	.	11	DD
**1143**	Polypay	Idaho 2004	3	.	11	ID
**1144**	Polypay	Idaho 2004	4	5.3	11	DD
**1145**	Polypay	Idaho 2004	5	699	11	II
**1146**	Polypay	Idaho 2004	6	4.9	11	II
**1147**	Polypay	Idaho 2004	5	959	11	ID
**1148**	Polypay	Idaho 2004	5	.	11	II
**1149**	Polypay	Idaho 2004	6	40	11	II
**1150**	Polypay	Idaho 2004	4	.	11	II
**1151**	Polypay	Idaho 2004	4	92	.	ID
**1152**	Polypay	Idaho 2004	4	..	11	ID

**1153**	Polypay	Idaho 2004	4	100	11	ID
**1154**	Polypay	Idaho 2004	3	.	11	ID
**1155**	Polypay	Idaho 2004	3	40	13	ID
**1156**	Polypay	Idaho 2004	5	1790	11	ID
**1157**	Polypay	Idaho 2004	5	.	11	ID
**1158**	Polypay	Idaho 2004	5	.	11	II
**1159**	Polypay	Idaho 2004	4	.	11	ID
**1160**	Polypay	Idaho 2004	4	.	11	DD
**1161**	Polypay	Idaho 2004	3	33	11	II
**1162**	Polypay	Idaho 2004	5	.	11	II
**1163**	Polypay	Idaho 2004	4	59	11	ID
**1164**	Polypay	Idaho 2004	4	.	.	ID
**1165**	Rambouillet	Idaho 2004	5	4210	11	ID
**1166**	Rambouillet	Idaho 2004	4	419	11	II
**1167**	Rambouillet	Idaho 2004	5	.	11	II
**1168**	Rambouillet	Idaho 2004	6	.	11	ID
**1169**	Rambouillet	Idaho 2004	6	262	11	II
**1170**	Rambouillet	Idaho 2004	5	3	11	DD
**1171**	Rambouillet	Idaho 2004	3	.	11	DD
**1172**	Rambouillet	Idaho 2004	4	.	.	II
**1173**	Rambouillet	Idaho 2004	5	148	11	ID
**1174**	Rambouillet	Idaho 2004	4	262	11	ID
**1175**	Rambouillet	Idaho 2004	4	17	11	DD
**1176**	Rambouillet	Idaho 2004	4	.	11	ID
**1177**	Rambouillet	Idaho 2004	4	2	11	ID
**1178**	Rambouillet	Idaho 2004	4	36	11	DD
**1179**	Rambouillet	Idaho 2004	5	6	11	ID
**1180**	Rambouillet	Idaho 2004	4	72	11	DD
**1181**	Rambouillet	Idaho 2004	5	.	11	II
**1182**	Rambouillet	Idaho 2004	3	222	11	ID
**1183**	Rambouillet	Idaho 2004	5	.	.	II
**1184**	Rambouillet	Idaho 2004	5	.	11	II
**1185**	Rambouillet	Idaho 2004	6	.	11	II
**1186**	Rambouillet	Idaho 2004	6	134	11	DD
**1187**	Rambouillet	Idaho 2004	5	255	.	II
**1188**	Rambouillet	Idaho 2004	4	330	.	II
**1189**	Rambouillet	Idaho 2004	4	13	11	ID
**1190**	Rambouillet	Idaho 2004	3	202	13	II
**1191**	Rambouillet	Idaho 2004	6	2430	12	II
**1192**	Rambouillet	Idaho 2004	4	.	11	DD
**1193**	Rambouillet	Idaho 2004	4	.	11	ID
**1194**	Rambouillet	Idaho 2004	4	67	11	II
**1195**	Rambouillet	Idaho 2004	5	2	11	DD

**1196**	Rambouillet	Idaho 2004	6	.	11	II
**1197**	Rambouillet	Idaho 2004	3	.	11	II
**1198**	Rambouillet	Idaho 2004	5	.	11	II
**1199**	Rambouillet	Idaho 2004	5	3	11	II
**1200**	Rambouillet	Idaho 2004	6	23	11	II
**1201**	Rambouillet	Idaho 2004	4	191	11	DD
**1202**	Rambouillet	Idaho 2004	3	89	11	ID
**1203**	Rambouillet	Idaho 2004	3	239	11	II
**1204**	Rambouillet	Idaho 2004	3	242	11	ID
**1205**	Rambouillet	Idaho 2004	3	298	110	ID
**1206**	Rambouillet	Idaho 2004	3	.	13	II
**1207**	Rambouillet	Idaho 2004	4	1010	11	ID
**1208**	Rambouillet	Idaho 2004	5	.	11	II
**1209**	Rambouillet	Idaho 2004	3	188	12	II
**1210**	Rambouillet	Idaho 2004	5	73	.	ID
**1211**	Rambouillet	Idaho 2004	3	2	11	ID
**1212**	Rambouillet	Idaho 2004	5	875	11	ID
**1213**	Rambouillet	Idaho 2004	5	1560	11	II
**1214**	Rambouillet	Idaho 2004	5	.	11	ID
**1215**	Rambouillet	Idaho 2004	5	.	11	ID
**1216**	Rambouillet	Idaho 2004	4	1700	33	ID
**1217**	Rambouillet	Idaho 2004	4	.	11	II
**1218**	Rambouillet	Idaho 2004	6	48	11	II
**1219**	Rambouillet	Idaho 2004	3	.	11	II
**1220**	Rambouillet	Idaho 2004	4	.	11	DD
**1221**	Rambouillet	Idaho 2004	5	4	110	II
**1222**	Rambouillet	Idaho 2004	6	65	13	II
**1223**	Rambouillet	Idaho 2004	6	.	11	II
**1224**	Rambouillet	Idaho 2004	3	.	11	II
**1225**	Rambouillet	Idaho 2004	3	215	11	ID
**1226**	Rambouillet	Idaho 2004	4	228	11	ID
**1227**	Rambouillet	Idaho 2004	6	.	11	II
**1228**	Rambouillet	Idaho 2004	4	1240	11	ID
**1229**	Rambouillet	Idaho 2004	3	1530	12	ID
**1230**	Rambouillet	Idaho 2004	4	435	.	II
**1231**	Rambouillet	Idaho 2004	5	.	11	ID
**1232**	Rambouillet	Idaho 2004	4	221	11	II
**1233**	Rambouillet	Idaho 2004	6	110	11	II
**1234**	Rambouillet	Idaho 2004	6	.	11	ID
**1235**	Rambouillet	Idaho 2004	6	.	11	ID
**1236**	Rambouillet	Idaho 2004	3	.	11	II
**1237**	Rambouillet	Idaho 2004	3	.	.	ID
**1238**	Rambouillet	Idaho 2004	6	13	.	ID

**1239**	Rambouillet	Idaho 2004	5	.	11	ID
**1240**	Rambouillet	Idaho 2004	5	.	11	ID
**1241**	Rambouillet	Idaho 2004	3	343	11	II
**1242**	Rambouillet	Idaho 2004	3	9	11	II
**1243**	Rambouillet	Idaho 2004	4	295	.	ID
**1244**	Rambouillet	Idaho 2004	3	.	11	II
**1245**	Rambouillet	Idaho 2004	4	.	11	II
**1246**	Rambouillet	Idaho 2004	6	143	11	II
**1247**	Rambouillet	Idaho 2004	4	12	11	II
**1248**	Rambouillet	Idaho 2004	5	.	11	DD
**1249**	Rambouillet	Idaho 2004	4	58	11	ID
**1250**	Rambouillet	Idaho 2004	5	.	11	II
**1251**	Rambouillet	Idaho 2004	5	2	11	ID
**1252**	Rambouillet	Idaho 2004	4	.	11	II
**1253**	Rambouillet	Idaho 2004	6	11	13	II
**1254**	Rambouillet	Idaho 2004	3	.	11	ID
**1255**	Rambouillet	Idaho 2004	4	141	13	II
**1256**	Rambouillet	Idaho 2004	3	78	11	II
**1257**	Rambouillet	Idaho 2004	5	87	11	II
**1258**	Rambouillet	Idaho 2004	5	.	.	II
**1259**	Rambouillet	Idaho 2004	6	.	.	II
**1260**	Rambouillet	Idaho 2004	3	.	12	II
**1261**	Rambouillet	Idaho 2004	6	35	11	II
**1262**	Rambouillet	Idaho 2004	5	.	.	II
**1263**	Rambouillet	Idaho 2004	3	2920	13	ID
**1264**	Rambouillet	Idaho 2004	3	.	11	II
**1265**	Rambouillet	Idaho 2004	3	504	.	II
**1266**	Rambouillet	Idaho 2004	3	66	11	ID
**1267**	Rambouillet	Idaho 2004	5	45	11	ID
**1268**	Rambouillet	Idaho 2004	4	.	11	ID
**1269**	Rambouillet	Idaho 2004	5	.	11	II
**1270**	Rambouillet	Idaho 2004	4	69	.	ID
**1271**	Rambouillet	Idaho 2004	5	.	11	II
**1272**	Rambouillet	Idaho 2004	5	3620	11	DD
**1273**	Rambouillet	Idaho 2004	6	23	11	II
**1274**	Rambouillet	Idaho 2004	3	2600	11	ID
**1275**	Rambouillet	Idaho 2004	6	.	11	II
**1276**	Rambouillet	Idaho 2004	5	1910	11	ID
**1277**	Rambouillet	Idaho 2004	4	114	13	ID
**1278**	Rambouillet	Idaho 2004	5	35	11	DD
**1279**	Rambouillet	Idaho 2004	3	13	.	II
**1280**	Rambouillet	Idaho 2004	4	512	11	ID
**1281**	Rambouillet	Idaho 2004	6	22	11	II

**1282**	Rambouillet	Idaho 2004	6	.	11	II
**1283**	Rambouillet	Idaho 2004	3	.	11	II
**1284**	Rambouillet	Idaho 2004	3	.	11	ID
**1285**	Rambouillet	Idaho 2004	4	1	11	ID
**1286**	Rambouillet	Idaho 2004	6	.	11	ID
**1287**	Rambouillet	Idaho 2004	3	26	13	II
**1288**	Rambouillet	Idaho 2004	4	.	11	ID
**1289**	Rambouillet	Idaho 2004	4	419	.	II
**1290**	Columbia	Idaho 2008	3	.	11	DD
**1291**	Columbia	Idaho 2008	5	.	11	ID
**1292**	Columbia	Idaho 2008	1	.	11	ID
**1293**	Columbia	Idaho 2008	1	.	11	II
**1294**	Columbia	Idaho 2008	1	.	11	II
**1295**	Columbia	Idaho 2008	2	.	11	ID
**1296**	Columbia	Idaho 2008	3	.	11	ID
**1297**	Columbia	Idaho 2008	3	.	11	ID
**1298**	Columbia	Idaho 2008	3	.	11	DD
**1299**	Columbia	Idaho 2008	3	.	11	ID
**1300**	Columbia	Idaho 2008	3	.	11	DD
**1301**	Columbia	Idaho 2008	3	.	11	.
**1302**	Columbia	Idaho 2008	3	.	11	ID
**1303**	Columbia	Idaho 2008	3	.	11	II
**1304**	Columbia	Idaho 2008	3	.	11	II
**1305**	Columbia	Idaho 2008	3	.	11	ID
**1306**	Columbia	Idaho 2008	3	.	11	II
**1307**	Columbia	Idaho 2008	3	.	11	ID
**1308**	Columbia	Idaho 2008	1	.	11	DD
**1309**	Columbia	Idaho 2008	4	.	11	ID
**1310**	Columbia	Idaho 2008	4	.	11	II
**1311**	Columbia	Idaho 2008	4	.	11	II
**1312**	Columbia	Idaho 2008	5	.	11	ID
**1313**	Columbia	Idaho 2008	5	.	11	ID
**1314**	Columbia	Idaho 2008	4	.	11	II
**1315**	Columbia	Idaho 2008	1	.	11	II
**1316**	Columbia	Idaho 2008	1	.	11	DD
**1317**	Columbia	Idaho 2008	1	.	11	ID
**1318**	Columbia	Idaho 2008	1	.	11	II
**1319**	Columbia	Idaho 2008	1	.	11	II
**1320**	Columbia	Idaho 2008	1	.	11	DD
**1321**	Columbia	Idaho 2008	1	.	11	DD
**1322**	Columbia	Idaho 2008	1	.	11	DD
**1323**	Columbia	Idaho 2008	1	.	11	ID
**1324**	Columbia	Idaho 2008	1	.	11	DD

**1325**	Columbia	Idaho 2008	1	.	11	ID
**1326**	Columbia	Idaho 2008	1	.	11	II
**1327**	Columbia	Idaho 2008	1	.	11	DD
**1328**	Columbia	Idaho 2008	1	.	11	ID
**1329**	Columbia	Idaho 2008	1	.	11	ID
**1330**	Columbia	Idaho 2008	1	.	11	ID
**1331**	Columbia	Idaho 2008	1	.	11	ID
**1332**	Columbia	Idaho 2008	1	.	11	DD
**1333**	Columbia	Idaho 2008	1	.	11	ID
**1334**	Columbia	Idaho 2008	1	.	11	II
**1335**	Columbia	Idaho 2008	1	.	11	DD
**1336**	Columbia	Idaho 2008	1	.	11	II
**1337**	Columbia	Idaho 2008	1	.	11	ID
**1338**	Columbia	Idaho 2008	1	.	11	DD
**1339**	Columbia	Idaho 2008	1	.	11	ID
**1340**	Columbia	Idaho 2008	1	.	11	DD
**1341**	Columbia	Idaho 2008	1	.	11	ID
**1342**	Columbia	Idaho 2008	1	.	11	ID
**1343**	Columbia	Idaho 2008	1	.	11	II
**1344**	Columbia	Idaho 2008	1	.	11	DD
**1345**	Columbia	Idaho 2008	1	.	11	ID
**1346**	Columbia	Idaho 2008	1	.	11	ID
**1347**	Columbia	Idaho 2008	1	.	11	ID
**1348**	Columbia	Idaho 2008	1	.	11	ID
**1349**	Columbia	Idaho 2008	1	.	11	ID
**1350**	Columbia	Idaho 2008	1	.	11	II
**1351**	Columbia	Idaho 2008	1	.	11	ID
**1352**	Columbia	Idaho 2008	1	.	11	ID
**1353**	Columbia	Idaho 2008	1	.	11	DD
**1354**	Columbia	Idaho 2008	1	.	11	ID
**1355**	Columbia	Idaho 2008	2	.	11	ID
**1356**	Columbia	Idaho 2008	2	.	11	II
**1357**	Columbia	Idaho 2008	2	.	11	II
**1358**	Columbia	Idaho 2008	2	.	11	II
**1359**	Columbia	Idaho 2008	2	.	11	ID
**1360**	Columbia	Idaho 2008	2	.	11	II
**1361**	Columbia	Idaho 2008	2	.	11	II
**1362**	Columbia	Idaho 2008	2	.	11	ID
**1363**	Columbia	Idaho 2008	2	.	11	DD
**1364**	Columbia	Idaho 2008	2	.	11	II
**1365**	Columbia	Idaho 2008	2	.	11	ID
**1366**	Columbia	Idaho 2008	2	.	11	ID
**1367**	Columbia	Idaho 2008	2	.	11	II

**1368**	Columbia	Idaho 2008	2	.	11	II
**1369**	Columbia	Idaho 2008	1	.	13	II
**1370**	Columbia	Idaho 2008	5	106	11	ID
**1371**	Columbia	Idaho 2008	5	109	11	ID
**1372**	Columbia	Idaho 2008	2	113	11	II
**1373**	Columbia	Idaho 2008	2	1140	11	II
**1374**	Columbia	Idaho 2008	2	12	11	DD
**1375**	Columbia	Idaho 2008	3	149	11	ID
**1376**	Columbia	Idaho 2008	3	15.4	11	II
**1377**	Columbia	Idaho 2008	2	15.6	11	ID
**1378**	Columbia	Idaho 2008	5	155	11	II
**1379**	Columbia	Idaho 2008	3	1620	11	II
**1380**	Columbia	Idaho 2008	4	17.8	11	II
**1381**	Columbia	Idaho 2008	3	17100	11	ID
**1382**	Columbia	Idaho 2008	4	1870	11	DD
**1383**	Columbia	Idaho 2008	3	188	11	II
**1384**	Columbia	Idaho 2008	4	19.2	11	DD
**1385**	Columbia	Idaho 2008	5	192	11	ID
**1386**	Columbia	Idaho 2008	3	20.5	11	II
**1387**	Columbia	Idaho 2008	5	205	11	.
**1388**	Columbia	Idaho 2008	4	2210	11	ID
**1389**	Columbia	Idaho 2008	1	24	11	DD
**1390**	Columbia	Idaho 2008	5	24	11	ID
**1391**	Columbia	Idaho 2008	5	266	11	II
**1392**	Columbia	Idaho 2008	2	28.9	11	II
**1393**	Columbia	Idaho 2008	3	285	11	ID
**1394**	Columbia	Idaho 2008	3	3.16	11	II
**1395**	Columbia	Idaho 2008	1	3.73	11	ID
**1396**	Columbia	Idaho 2008	2	304	11	II
**1397**	Columbia	Idaho 2008	4	309	11	II
**1398**	Columbia	Idaho 2008	3	33500	11	II
**1399**	Columbia	Idaho 2008	2	344	11	II
**1400**	Columbia	Idaho 2008	3	345	11	ID
**1401**	Columbia	Idaho 2008	3	358	11	ID
**1402**	Columbia	Idaho 2008	4	378	11	DD
**1403**	Columbia	Idaho 2008	2	443	11	ID
**1404**	Columbia	Idaho 2008	3	470	11	II
**1405**	Columbia	Idaho 2008	3	48100	11	II
**1406**	Columbia	Idaho 2008	3	4900	11	ID
**1407**	Columbia	Idaho 2008	2	5160	11	ID
**1408**	Columbia	Idaho 2008	4	51900	11	II
**1409**	Columbia	Idaho 2008	2	5420	11	II
**1410**	Columbia	Idaho 2008	3	57.9	11	ID

**1411**	Columbia	Idaho 2008	2	576	11	II
**1412**	Columbia	Idaho 2008	4	586	11	II
**1413**	Columbia	Idaho 2008	3	61.3	11	ID
**1414**	Columbia	Idaho 2008	4	677	11	DD
**1415**	Columbia	Idaho 2008	3	696	11	ID
**1416**	Columbia	Idaho 2008	4	7.71	11	II
**1417**	Columbia	Idaho 2008	3	732	11	II
**1418**	Columbia	Idaho 2008	4	776	11	II
**1419**	Columbia	Idaho 2008	2	785	11	DD
**1420**	Columbia	Idaho 2008	4	80.5	11	II
**1421**	Columbia	Idaho 2008	3	829	11	DD
**1422**	Columbia	Idaho 2008	1	851	11	ID
**1423**	Columbia	Idaho 2008	3	9.14	.	ID
**1424**	Columbia	Idaho 2008	3	9.84	11	ID
**1425**	Columbia	Idaho 2008	1	93	11	II
**1426**	Columbia	Idaho 2008	2	945	11	ID
**1427**	Columbia	Idaho 2008	2	99.3	11	ID
**1428**	Polypay	Idaho 2008	1	.	11	.
**1429**	Polypay	Idaho 2008	1	.	11	II
**1430**	Polypay	Idaho 2008	1	.	11	II
**1431**	Polypay	Idaho 2008	1	.	11	.
**1432**	Polypay	Idaho 2008	1	.	11	II
**1433**	Polypay	Idaho 2008	1	.	11	ID
**1434**	Polypay	Idaho 2008	2	.	.	.
**1435**	Polypay	Idaho 2008	3	.	11	ID
**1436**	Polypay	Idaho 2008	1	.	11	ID
**1437**	Polypay	Idaho 2008	3	.	11	ID
**1438**	Polypay	Idaho 2008	3	.	11	II
**1439**	Polypay	Idaho 2008	1	.	11	ID
**1440**	Polypay	Idaho 2008	1	.	11	ID
**1441**	Polypay	Idaho 2008	1	.	11	II
**1442**	Polypay	Idaho 2008	1	.	11	II
**1443**	Polypay	Idaho 2008	1	.	11	ID
**1444**	Polypay	Idaho 2008	1	.	11	ID
**1445**	Polypay	Idaho 2008	1	.	11	II
**1446**	Polypay	Idaho 2008	1	.	11	II
**1447**	Polypay	Idaho 2008	1	.	11	ID
**1448**	Polypay	Idaho 2008	1	.	11	ID
**1449**	Polypay	Idaho 2008	1	.	11	DD
**1450**	Polypay	Idaho 2008	1	.	11	II
**1451**	Polypay	Idaho 2008	1	.	11	ID
**1452**	Polypay	Idaho 2008	1	.	11	ID
**1453**	Polypay	Idaho 2008	1	.	11	ID

**1454**	Polypay	Idaho 2008	1	.	11	II
**1455**	Polypay	Idaho 2008	1	.	11	II
**1456**	Polypay	Idaho 2008	1	.	11	DD
**1457**	Polypay	Idaho 2008	1	.	11	II
**1458**	Polypay	Idaho 2008	1	.	11	ID
**1459**	Polypay	Idaho 2008	1	.	11	ID
**1460**	Polypay	Idaho 2008	1	.	11	ID
**1461**	Polypay	Idaho 2008	3	.	11	II
**1462**	Polypay	Idaho 2008	1	.	11	ID
**1463**	Polypay	Idaho 2008	1	.	11	ID
**1464**	Polypay	Idaho 2008	1	.	11	II
**1465**	Polypay	Idaho 2008	3	.	11	ID
**1466**	Polypay	Idaho 2008	1	.	11	ID
**1467**	Polypay	Idaho 2008	3	.	11	ID
**1468**	Polypay	Idaho 2008	3	.	11	DD
**1469**	Polypay	Idaho 2008	3	.	11	ID
**1470**	Polypay	Idaho 2008	3	.	11	II
**1471**	Polypay	Idaho 2008	3	.	11	II
**1472**	Polypay	Idaho 2008	3	.	11	II
**1473**	Polypay	Idaho 2008	1	.	11	ID
**1474**	Polypay	Idaho 2008	1	.	11	II
**1475**	Polypay	Idaho 2008	3	.	11	ID
**1476**	Polypay	Idaho 2008	1	.	11	II
**1477**	Polypay	Idaho 2008	1	.	11	DD
**1478**	Polypay	Idaho 2008	3	.	11	ID
**1479**	Polypay	Idaho 2008	3	.	11	ID
**1480**	Polypay	Idaho 2008	3	.	11	ID
**1481**	Polypay	Idaho 2008	3	.	11	ID
**1482**	Polypay	Idaho 2008	3	.	11	ID
**1483**	Polypay	Idaho 2008	1	.	11	.
**1484**	Polypay	Idaho 2008	3	.	11	.
**1485**	Polypay	Idaho 2008	1	.	11	ID
**1486**	Polypay	Idaho 2008	1	.	11	DD
**1487**	Polypay	Idaho 2008	3	.	11	II
**1488**	Polypay	Idaho 2008	2	.	11	DD
**1489**	Polypay	Idaho 2008	3	.	11	ID
**1490**	Polypay	Idaho 2008	3	.	11	.
**1491**	Polypay	Idaho 2008	1	.	11	II
**1492**	Polypay	Idaho 2008	1	.	11	II
**1493**	Polypay	Idaho 2008	3	.	11	ID
**1494**	Polypay	Idaho 2008	3	.	11	II
**1495**	Polypay	Idaho 2008	3	.	11	II
**1496**	Polypay	Idaho 2008	1	.	11	ID

**1497**	Polypay	Idaho 2008	3	.	11	ID
**1498**	Polypay	Idaho 2008	3	.	11	II
**1499**	Polypay	Idaho 2008	3	.	11	II
**1500**	Polypay	Idaho 2008	3	.	11	II
**1501**	Polypay	Idaho 2008	1	.	11	ID
**1502**	Polypay	Idaho 2008	1	.	11	ID
**1503**	Polypay	Idaho 2008	1	.	11	II
**1504**	Polypay	Idaho 2008	3	.	11	ID
**1505**	Polypay	Idaho 2008	1	.	11	II
**1506**	Polypay	Idaho 2008	1	.	11	II
**1507**	Polypay	Idaho 2008	3	.	11	II
**1508**	Polypay	Idaho 2008	3	.	11	II
**1509**	Polypay	Idaho 2008	1	.	11	ID
**1510**	Polypay	Idaho 2008	3	.	11	ID
**1511**	Polypay	Idaho 2008	3	.	11	II
**1512**	Polypay	Idaho 2008	3	.	11	II
**1513**	Polypay	Idaho 2008	3	.	11	II
**1514**	Polypay	Idaho 2008	3	.	11	ID
**1515**	Polypay	Idaho 2008	1	.	11	ID
**1516**	Polypay	Idaho 2008	1	.	11	II
**1517**	Polypay	Idaho 2008	1	.	11	ID
**1518**	Polypay	Idaho 2008	3	.	11	II
**1519**	Polypay	Idaho 2008	3	.	11	ID
**1520**	Polypay	Idaho 2008	3	.	11	.
**1521**	Polypay	Idaho 2008	3	.	11	.
**1522**	Polypay	Idaho 2008	3	.	11	.
**1523**	Polypay	Idaho 2008	3	.	11	II
**1524**	Polypay	Idaho 2008	1	.	11	ID
**1525**	Polypay	Idaho 2008	3	.	11	II
**1526**	Polypay	Idaho 2008	3	.	11	.
**1527**	Polypay	Idaho 2008	3	.	11	.
**1528**	Polypay	Idaho 2008	1	.	11	ID
**1529**	Polypay	Idaho 2008	1	.	11	II
**1530**	Polypay	Idaho 2008	3	.	11	ID
**1531**	Polypay	Idaho 2008	3	.	11	ID
**1532**	Polypay	Idaho 2008	1	.	11	DD
**1533**	Polypay	Idaho 2008	3	.	11	II
**1534**	Polypay	Idaho 2008	3	.	11	.
**1535**	Polypay	Idaho 2008	4	.	11	ID
**1536**	Polypay	Idaho 2008	4	.	11	II
**1537**	Polypay	Idaho 2008	4	.	11	II
**1538**	Polypay	Idaho 2008	4	.	11	ID
**1539**	Polypay	Idaho 2008	4	.	11	II

**1540**	Polypay	Idaho 2008	1	.	11	.
**1541**	Polypay	Idaho 2008	4	.	11	ID
**1542**	Polypay	Idaho 2008	4	.	11	ID
**1543**	Polypay	Idaho 2008	4	.	11	II
**1544**	Polypay	Idaho 2008	4	.	11	DD
**1545**	Polypay	Idaho 2008	4	.	11	II
**1546**	Polypay	Idaho 2008	4	.	11	II
**1547**	Polypay	Idaho 2008	4	.	11	II
**1548**	Polypay	Idaho 2008	5	.	11	II
**1549**	Polypay	Idaho 2008	5	.	11	II
**1550**	Polypay	Idaho 2008	5	.	11	II
**1551**	Polypay	Idaho 2008	5	.	11	II
**1552**	Polypay	Idaho 2008	1	.	11	.
**1553**	Polypay	Idaho 2008	1	.	11	.
**1554**	Polypay	Idaho 2008	1	.	11	.
**1555**	Polypay	Idaho 2008	1	.	11	.
**1556**	Polypay	Idaho 2008	1	.	11	.
**1557**	Polypay	Idaho 2008	1	.	11	.
**1558**	Polypay	Idaho 2008	1	.	11	II
**1559**	Polypay	Idaho 2008	1	.	11	II
**1560**	Polypay	Idaho 2008	1	.	11	.
**1561**	Polypay	Idaho 2008	1	.	11	II
**1562**	Polypay	Idaho 2008	1	.	11	.
**1563**	Polypay	Idaho 2008	1	.	11	.
**1564**	Polypay	Idaho 2008	1	.	11	.
**1565**	Polypay	Idaho 2008	1	.	11	II
**1566**	Polypay	Idaho 2008	1	.	11	.
**1567**	Polypay	Idaho 2008	1	.	11	.
**1568**	Polypay	Idaho 2008	1	.	11	II
**1569**	Polypay	Idaho 2008	2	.	11	II
**1570**	Polypay	Idaho 2008	1	.	11	II
**1571**	Polypay	Idaho 2008	1	.	11	II
**1572**	Polypay	Idaho 2008	1	.	11	II
**1573**	Polypay	Idaho 2008	1	.	11	DD
**1574**	Polypay	Idaho 2008	1	.	11	II
**1575**	Polypay	Idaho 2008	1	.	11	ID
**1576**	Polypay	Idaho 2008	1	.	11	ID
**1577**	Polypay	Idaho 2008	1	.	11	ID
**1578**	Polypay	Idaho 2008	2	.	11	II
**1579**	Polypay	Idaho 2008	2	.	11	ID
**1580**	Polypay	Idaho 2008	2	.	11	II
**1581**	Polypay	Idaho 2008	2	.	11	II
**1582**	Polypay	Idaho 2008	2	.	11	ID

**1583**	Polypay	Idaho 2008	2	.	11	II
**1584**	Polypay	Idaho 2008	2	.	11	DD
**1585**	Polypay	Idaho 2008	1	.	11	II
**1586**	Polypay	Idaho 2008	1	.	11	DD
**1587**	Polypay	Idaho 2008	2	.	11	ID
**1588**	Polypay	Idaho 2008	2	.	11	II
**1589**	Polypay	Idaho 2008	2	.	11	II
**1590**	Polypay	Idaho 2008	1	.	11	ID
**1591**	Polypay	Idaho 2008	1	.	11	II
**1592**	Polypay	Idaho 2008	2	.	11	ID
**1593**	Polypay	Idaho 2008	1	.	11	II
**1594**	Polypay	Idaho 2008	2	.	11	II
**1595**	Polypay	Idaho 2008	1	.	11	DD
**1596**	Polypay	Idaho 2008	1	.	11	II
**1597**	Polypay	Idaho 2008	1	.	11	ID
**1598**	Polypay	Idaho 2008	1	.	11	.
**1599**	Polypay	Idaho 2008	2	.	11	II
**1600**	Polypay	Idaho 2008	2	.	11	ID
**1601**	Polypay	Idaho 2008	2	.	11	.
**1602**	Polypay	Idaho 2008	2	.	11	.
**1603**	Polypay	Idaho 2008	1	.	11	II
**1604**	Polypay	Idaho 2008	2	.	11	.
**1605**	Polypay	Idaho 2008	2	.	11	.
**1606**	Polypay	Idaho 2008	2	.	11	.
**1607**	Polypay	Idaho 2008	1	.	11	ID
**1608**	Polypay	Idaho 2008	1	.	11	II
**1609**	Polypay	Idaho 2008	1	.	11	II
**1610**	Polypay	Idaho 2008	1	.	11	ID
**1611**	Polypay	Idaho 2008	1	.	11	II
**1612**	Polypay	Idaho 2008	1	.	11	II
**1613**	Polypay	Idaho 2008	1	.	11	ID
**1614**	Polypay	Idaho 2008	1	.	11	ID
**1615**	Polypay	Idaho 2008	1	.	11	II
**1616**	Polypay	Idaho 2008	1	.	11	II
**1617**	Polypay	Idaho 2008	1	.	11	II
**1618**	Polypay	Idaho 2008	2	.	11	ID
**1619**	Polypay	Idaho 2008	1	.	11	II
**1620**	Polypay	Idaho 2008	1	.	11	.
**1621**	Polypay	Idaho 2008	1	.	11	II
**1622**	Polypay	Idaho 2008	1	.	11	II
**1623**	Polypay	Idaho 2008	1	.	11	II
**1624**	Polypay	Idaho 2008	1	.	11	.
**1625**	Polypay	Idaho 2008	1	.	11	II

**1626**	Polypay	Idaho 2008	1	.	11	.
**1627**	Polypay	Idaho 2008	1	.	11	DD
**1628**	Polypay	Idaho 2008	1	.	11	II
**1629**	Polypay	Idaho 2008	1	.	11	ID
**1630**	Polypay	Idaho 2008	2	.	11	ID
**1631**	Polypay	Idaho 2008	2	.	11	II
**1632**	Polypay	Idaho 2008	2	.	11	II
**1633**	Polypay	Idaho 2008	1	.	11	ID
**1634**	Polypay	Idaho 2008	1	.	11	ID
**1635**	Polypay	Idaho 2008	2	.	11	ID
**1636**	Polypay	Idaho 2008	2	.	11	ID
**1637**	Polypay	Idaho 2008	1	.	11	II
**1638**	Polypay	Idaho 2008	2	.	11	II
**1639**	Polypay	Idaho 2008	1	.	11	II
**1640**	Polypay	Idaho 2008	1	.	11	ID
**1641**	Polypay	Idaho 2008	1	.	11	ID
**1642**	Polypay	Idaho 2008	2	.	11	ID
**1643**	Polypay	Idaho 2008	1	.	11	DD
**1644**	Polypay	Idaho 2008	1	.	11	II
**1645**	Polypay	Idaho 2008	2	.	11	II
**1646**	Polypay	Idaho 2008	2	.	11	II
**1647**	Polypay	Idaho 2008	2	.	11	II
**1648**	Polypay	Idaho 2008	1	.	11	II
**1649**	Polypay	Idaho 2008	2	.	11	ID
**1650**	Polypay	Idaho 2008	1	.	11	ID
**1651**	Polypay	Idaho 2008	1	.	11	II
**1652**	Polypay	Idaho 2008	2	.	11	II
**1653**	Polypay	Idaho 2008	1	.	11	ID
**1654**	Polypay	Idaho 2008	1	.	11	II
**1655**	Polypay	Idaho 2008	2	.	11	II
**1656**	Polypay	Idaho 2008	2	.	11	II
**1657**	Polypay	Idaho 2008	1	.	11	ID
**1658**	Polypay	Idaho 2008	1	.	11	ID
**1659**	Polypay	Idaho 2008	2	.	11	II
**1660**	Polypay	Idaho 2008	2	.	11	II
**1661**	Polypay	Idaho 2008	1	.	11	II
**1662**	Polypay	Idaho 2008	2	.	11	II
**1663**	Polypay	Idaho 2008	2	.	11	II
**1664**	Polypay	Idaho 2008	2	.	11	ID
**1665**	Polypay	Idaho 2008	2	.	11	ID
**1666**	Polypay	Idaho 2008	1	.	11	II
**1667**	Polypay	Idaho 2008	1	.	12	II
**1668**	Polypay	Idaho 2008	1	.	12	ID

**1669**	Polypay	Idaho 2008	1	.	13	ID
**1670**	Polypay	Idaho 2008	1	.	13	II
**1671**	Polypay	Idaho 2008	1	.	13	II
**1672**	Polypay	Idaho 2008	1	.	13	ID
**1673**	Polypay	Idaho 2008	4	.	13	ID
**1674**	Polypay	Idaho 2008	1	.	13	II
**1675**	Polypay	Idaho 2008	1	.	13	DD
**1676**	Polypay	Idaho 2008	1	.	13	ID
**1677**	Polypay	Idaho 2008	1	.	14	II
**1678**	Polypay	Idaho 2008	2	10.8	11	ID
**1679**	Polypay	Idaho 2008	4	10.9	13	.
**1680**	Polypay	Idaho 2008	3	1010	13	II
**1681**	Polypay	Idaho 2008	1	102	13	II
**1682**	Polypay	Idaho 2008	5	103	11	ID
**1683**	Polypay	Idaho 2008	3	105	33	II
**1684**	Polypay	Idaho 2008	4	105	33	.
**1685**	Polypay	Idaho 2008	4	1050	11	.
**1686**	Polypay	Idaho 2008	4	1080	11	.
**1687**	Polypay	Idaho 2008	5	11.2	11	II
**1688**	Polypay	Idaho 2008	2	118	11	II
**1689**	Polypay	Idaho 2008	4	12.3	11	ID
**1690**	Polypay	Idaho 2008	2	121	11	.
**1691**	Polypay	Idaho 2008	4	124	11	II
**1692**	Polypay	Idaho 2008	2	127	11	II
**1693**	Polypay	Idaho 2008	4	13	11	II
**1694**	Polypay	Idaho 2008	4	132	13	DD
**1695**	Polypay	Idaho 2008	4	13300	11	.
**1696**	Polypay	Idaho 2008	1	134	13	ID
**1697**	Polypay	Idaho 2008	4	135	11	II
**1698**	Polypay	Idaho 2008	4	136	11	II
**1699**	Polypay	Idaho 2008	3	139	11	II
**1700**	Polypay	Idaho 2008	5	14.2	11	.
**1701**	Polypay	Idaho 2008	3	14.9	13	.
**1702**	Polypay	Idaho 2008	2	140	11	DD
**1703**	Polypay	Idaho 2008	4	150	11	II
**1704**	Polypay	Idaho 2008	3	151	11	ID
**1705**	Polypay	Idaho 2008	1	153	13	ID
**1706**	Polypay	Idaho 2008	4	155	11	ID
**1707**	Polypay	Idaho 2008	4	156	11	ID
**1708**	Polypay	Idaho 2008	2	16.6	11	.
**1709**	Polypay	Idaho 2008	2	16.6	13	II
**1710**	Polypay	Idaho 2008	2	16.8	11	II
**1711**	Polypay	Idaho 2008	2	163	13	ID

**1712**	Polypay	Idaho 2008	3	1670	11	II
**1713**	Polypay	Idaho 2008	2	169	11	ID
**1714**	Polypay	Idaho 2008	4	16900	11	II
**1715**	Polypay	Idaho 2008	4	16900	11	ID
**1716**	Polypay	Idaho 2008	1	171	11	ID
**1717**	Polypay	Idaho 2008	3	17200	11	II
**1718**	Polypay	Idaho 2008	4	1740	11	II
**1719**	Polypay	Idaho 2008	2	1750	11	II
**1720**	Polypay	Idaho 2008	4	176	11	.
**1721**	Polypay	Idaho 2008	3	177	11	ID
**1722**	Polypay	Idaho 2008	2	181	11	ID
**1723**	Polypay	Idaho 2008	4	181	11	II
**1724**	Polypay	Idaho 2008	4	182	11	.
**1725**	Polypay	Idaho 2008	4	184	11	ID
**1726**	Polypay	Idaho 2008	4	188	11	II
**1727**	Polypay	Idaho 2008	5	1880	11	ID
**1728**	Polypay	Idaho 2008	2	189	13	II
**1729**	Polypay	Idaho 2008	3	191	11	II
**1730**	Polypay	Idaho 2008	2	192	11	II
**1731**	Polypay	Idaho 2008	2	2.06	11	ID
**1732**	Polypay	Idaho 2008	1	2.28	11	II
**1733**	Polypay	Idaho 2008	3	20.3	11	II
**1734**	Polypay	Idaho 2008	4	20.3	11	II
**1735**	Polypay	Idaho 2008	4	2070	11	ID
**1736**	Polypay	Idaho 2008	3	21.9	11	II
**1737**	Polypay	Idaho 2008	5	21000	11	II
**1738**	Polypay	Idaho 2008	3	2120	11	ID
**1739**	Polypay	Idaho 2008	2	216	11	.
**1740**	Polypay	Idaho 2008	1	226	11	II
**1741**	Polypay	Idaho 2008	4	23	11	ID
**1742**	Polypay	Idaho 2008	4	23.9	11	ID
**1743**	Polypay	Idaho 2008	1	236	11	II
**1744**	Polypay	Idaho 2008	5	23800	11	.
**1745**	Polypay	Idaho 2008	2	240	13	II
**1746**	Polypay	Idaho 2008	2	2410	11	ID
**1747**	Polypay	Idaho 2008	3	244	11	ID
**1748**	Polypay	Idaho 2008	3	25.1	11	ID
**1749**	Polypay	Idaho 2008	3	2510	11	II
**1750**	Polypay	Idaho 2008	4	253	11	II
**1751**	Polypay	Idaho 2008	2	266	11	.
**1752**	Polypay	Idaho 2008	4	270	11	II
**1753**	Polypay	Idaho 2008	3	28.1	11	II
**1754**	Polypay	Idaho 2008	1	2800	11	II

**1755**	Polypay	Idaho 2008	3	287	11	.
**1756**	Polypay	Idaho 2008	4	293	11	II
**1757**	Polypay	Idaho 2008	2	2930	11	II
**1758**	Polypay	Idaho 2008	2	3.19	11	II
**1759**	Polypay	Idaho 2008	1	3.21	11	ID
**1760**	Polypay	Idaho 2008	4	3.37	13	ID
**1761**	Polypay	Idaho 2008	3	30.7	11	II
**1762**	Polypay	Idaho 2008	1	3120	11	II
**1763**	Polypay	Idaho 2008	2	3140	11	ID
**1764**	Polypay	Idaho 2008	3	33	11	.
**1765**	Polypay	Idaho 2008	2	3350	11	II
**1766**	Polypay	Idaho 2008	2	337	11	II
**1767**	Polypay	Idaho 2008	4	35.2	11	.
**1768**	Polypay	Idaho 2008	1	36.9	11	.
**1769**	Polypay	Idaho 2008	1	3600	11	ID
**1770**	Polypay	Idaho 2008	4	3690	11	II
**1771**	Polypay	Idaho 2008	5	379	11	II
**1772**	Polypay	Idaho 2008	5	396	11	II
**1773**	Polypay	Idaho 2008	3	398	11	II
**1774**	Polypay	Idaho 2008	2	4.41	11	ID
**1775**	Polypay	Idaho 2008	2	4.56	11	II
**1776**	Polypay	Idaho 2008	2	409	13	DD
**1777**	Polypay	Idaho 2008	3	4170	11	DD
**1778**	Polypay	Idaho 2008	5	436	11	II
**1779**	Polypay	Idaho 2008	2	44.3	11	ID
**1780**	Polypay	Idaho 2008	3	45.3	11	II
**1781**	Polypay	Idaho 2008	4	456	11	II
**1782**	Polypay	Idaho 2008	2	4850	11	II
**1783**	Polypay	Idaho 2008	2	5.39	13	II
**1784**	Polypay	Idaho 2008	2	5.61	11	.
**1785**	Polypay	Idaho 2008	2	50.1	11	II
**1786**	Polypay	Idaho 2008	5	54.2	11	.
**1787**	Polypay	Idaho 2008	2	541	13	.
**1788**	Polypay	Idaho 2008	1	55.4	11	ID
**1789**	Polypay	Idaho 2008	2	55.6	12	II
**1790**	Polypay	Idaho 2008	4	5670	11	II
**1791**	Polypay	Idaho 2008	4	58.9	11	II
**1792**	Polypay	Idaho 2008	4	59.1	11	DD
**1793**	Polypay	Idaho 2008	1	5900	14	ID
**1794**	Polypay	Idaho 2008	2	6.19	13	DD
**1795**	Polypay	Idaho 2008	4	606	11	ID
**1796**	Polypay	Idaho 2008	1	61.1	11	ID
**1797**	Polypay	Idaho 2008	3	630	11	.

**1798**	Polypay	Idaho 2008	3	652	11	II
**1799**	Polypay	Idaho 2008	3	672	11	ID
**1800**	Polypay	Idaho 2008	4	69.9	11	ID
**1801**	Polypay	Idaho 2008	5	7.09	11	II
**1802**	Polypay	Idaho 2008	4	7.4	11	II
**1803**	Polypay	Idaho 2008	4	7.51	11	II
**1804**	Polypay	Idaho 2008	4	7.91	13	DD
**1805**	Polypay	Idaho 2008	1	7.99	11	II
**1806**	Polypay	Idaho 2008	2	7110	13	ID
**1807**	Polypay	Idaho 2008	2	75.7	11	ID
**1808**	Polypay	Idaho 2008	3	77.2	11	ID
**1809**	Polypay	Idaho 2008	3	78.1	11	.
**1810**	Polypay	Idaho 2008	5	785	11	ID
**1811**	Polypay	Idaho 2008	2	8.44	13	ID
**1812**	Polypay	Idaho 2008	3	80.2	11	.
**1813**	Polypay	Idaho 2008	2	81	11	II
**1814**	Polypay	Idaho 2008	1	829	11	II
**1815**	Polypay	Idaho 2008	1	86.4	12	ID
**1816**	Polypay	Idaho 2008	2	87.2	11	II
**1817**	Polypay	Idaho 2008	2	87.5	11	II
**1818**	Polypay	Idaho 2008	3	88.1	11	II
**1819**	Polypay	Idaho 2008	3	891	11	ID
**1820**	Polypay	Idaho 2008	1	9.35	11	.
**1821**	Polypay	Idaho 2008	1	91	11	.
**1822**	Polypay	Idaho 2008	3	981	11	ID
**1823**	Polypay	Idaho 2008	3	99.8	11	ID
**1824**	Polypay	Idaho 2008	1	.	11	ID
**1825**	Polypay	Idaho 2008	5	.	11	DD
**1826**	Polypay	Idaho 2008	1	.	11	DD
**1827**	Polypay	Idaho 2008	1	.	11	II
**1828**	Rambouillet	Idaho 2008	2	.	11	II
**1829**	Rambouillet	Idaho 2008	3	.	11	II
**1830**	Rambouillet	Idaho 2008	1	.	11	II
**1831**	Rambouillet	Idaho 2008	2	.	11	.
**1832**	Rambouillet	Idaho 2008	1	.	11	ID
**1833**	Rambouillet	Idaho 2008	1	.	11	II
**1834**	Rambouillet	Idaho 2008	2	.	11	II
**1835**	Rambouillet	Idaho 2008	1	.	11	DD
**1836**	Rambouillet	Idaho 2008	1	.	11	ID
**1837**	Rambouillet	Idaho 2008	1	.	11	.
**1838**	Rambouillet	Idaho 2008	3	.	11	DD
**1839**	Rambouillet	Idaho 2008	4	.	11	ID
**1840**	Rambouillet	Idaho 2008	4	.	11	II

**1841**	Rambouillet	Idaho 2008	1	.	11	II
**1842**	Rambouillet	Idaho 2008	4	.	11	II
**1843**	Rambouillet	Idaho 2008	1	.	11	ID
**1844**	Rambouillet	Idaho 2008	2	.	11	II
**1845**	Rambouillet	Idaho 2008	3	.	11	.
**1846**	Rambouillet	Idaho 2008	1	.	14	ID
**1847**	Rambouillet	Idaho 2008	4	.	11	.
**1848**	Rambouillet	Idaho 2008	4	.	11	II
**1849**	Rambouillet	Idaho 2008	4	.	11	II
**1850**	Rambouillet	Idaho 2008	4	.	11	II
**1851**	Rambouillet	Idaho 2008	3	.	11	ID
**1852**	Rambouillet	Idaho 2008	3	.	11	II
**1853**	Rambouillet	Idaho 2008	3	.	11	ID
**1854**	Rambouillet	Idaho 2008	3	.	11	ID
**1855**	Rambouillet	Idaho 2008	3	.	11	ID
**1856**	Rambouillet	Idaho 2008	4	.	11	ID
**1857**	Rambouillet	Idaho 2008	1	.	11	II
**1858**	Rambouillet	Idaho 2008	1	.	11	ID
**1859**	Rambouillet	Idaho 2008	1	.	11	II
**1860**	Rambouillet	Idaho 2008	1	.	11	II
**1861**	Rambouillet	Idaho 2008	1	.	11	ID
**1862**	Rambouillet	Idaho 2008	1	.	11	II
**1863**	Rambouillet	Idaho 2008	1	.	11	II
**1864**	Rambouillet	Idaho 2008	1	.	11	ID
**1865**	Rambouillet	Idaho 2008	1	.	11	ID
**1866**	Rambouillet	Idaho 2008	1	.	11	ID
**1867**	Rambouillet	Idaho 2008	1	.	11	ID
**1868**	Rambouillet	Idaho 2008	1	.	11	II
**1869**	Rambouillet	Idaho 2008	1	.	11	ID
**1870**	Rambouillet	Idaho 2008	1	.	11	II
**1871**	Rambouillet	Idaho 2008	1	.	11	II
**1872**	Rambouillet	Idaho 2008	1	.	11	II
**1873**	Rambouillet	Idaho 2008	1	.	11	ID
**1874**	Rambouillet	Idaho 2008	1	.	11	II
**1875**	Rambouillet	Idaho 2008	1	.	11	II
**1876**	Rambouillet	Idaho 2008	1	.	11	ID
**1877**	Rambouillet	Idaho 2008	1	.	11	II
**1878**	Rambouillet	Idaho 2008	1	.	11	.
**1879**	Rambouillet	Idaho 2008	1	.	11	.
**1880**	Rambouillet	Idaho 2008	1	.	11	.
**1881**	Rambouillet	Idaho 2008	1	.	11	.
**1882**	Rambouillet	Idaho 2008	1	.	11	II
**1883**	Rambouillet	Idaho 2008	1	.	11	.

**1884**	Rambouillet	Idaho 2008	3	.	11	ID
**1885**	Rambouillet	Idaho 2008	2	.	11	II
**1886**	Rambouillet	Idaho 2008	4	.	11	II
**1887**	Rambouillet	Idaho 2008	1	.	11	II
**1888**	Rambouillet	Idaho 2008	1	.	11	II
**1889**	Rambouillet	Idaho 2008	1	.	11	II
**1890**	Rambouillet	Idaho 2008	1	.	11	II
**1891**	Rambouillet	Idaho 2008	1	.	11	DD
**1892**	Rambouillet	Idaho 2008	1	.	11	ID
**1893**	Rambouillet	Idaho 2008	1	.	11	II
**1894**	Rambouillet	Idaho 2008	1	.	11	ID
**1895**	Rambouillet	Idaho 2008	1	.	11	II
**1896**	Rambouillet	Idaho 2008	5	.	11	II
**1897**	Rambouillet	Idaho 2008	1	.	11	II
**1898**	Rambouillet	Idaho 2008	5	.	11	II
**1899**	Rambouillet	Idaho 2008	1	.	11	ID
**1900**	Rambouillet	Idaho 2008	1	.	11	II
**1901**	Rambouillet	Idaho 2008	1	.	11	II
**1902**	Rambouillet	Idaho 2008	1	.	11	ID
**1903**	Rambouillet	Idaho 2008	1	.	11	II
**1904**	Rambouillet	Idaho 2008	3	.	11	II
**1905**	Rambouillet	Idaho 2008	4	.	11	ID
**1906**	Rambouillet	Idaho 2008	4	.	11	ID
**1907**	Rambouillet	Idaho 2008	4	.	11	ID
**1908**	Rambouillet	Idaho 2008	1	.	11	II
**1909**	Rambouillet	Idaho 2008	4	.	11	DD
**1910**	Rambouillet	Idaho 2008	4	.	11	II
**1911**	Rambouillet	Idaho 2008	1	.	11	II
**1912**	Rambouillet	Idaho 2008	4	.	11	ID
**1913**	Rambouillet	Idaho 2008	4	.	11	II
**1914**	Rambouillet	Idaho 2008	4	.	11	ID
**1915**	Rambouillet	Idaho 2008	4	.	11	II
**1916**	Rambouillet	Idaho 2008	4	.	11	II
**1917**	Rambouillet	Idaho 2008	4	.	11	II
**1918**	Rambouillet	Idaho 2008	4	.	11	II
**1919**	Rambouillet	Idaho 2008	4	.	11	ID
**1920**	Rambouillet	Idaho 2008	4	.	11	ID
**1921**	Rambouillet	Idaho 2008	4	.	11	ID
**1922**	Rambouillet	Idaho 2008	4	.	11	ID
**1923**	Rambouillet	Idaho 2008	4	.	11	ID
**1924**	Rambouillet	Idaho 2008	1	.	11	II
**1925**	Rambouillet	Idaho 2008	4	.	11	ID
**1926**	Rambouillet	Idaho 2008	4	.	11	II

**1927**	Rambouillet	Idaho 2008	1	.	11	II
**1928**	Rambouillet	Idaho 2008	1	.	11	DD
**1929**	Rambouillet	Idaho 2008	1	.	11	II
**1930**	Rambouillet	Idaho 2008	1	.	11	ID
**1931**	Rambouillet	Idaho 2008	1	.	11	ID
**1932**	Rambouillet	Idaho 2008	4	.	11	ID
**1933**	Rambouillet	Idaho 2008	4	.	11	ID
**1934**	Rambouillet	Idaho 2008	4	.	11	II
**1935**	Rambouillet	Idaho 2008	1	.	11	ID
**1936**	Rambouillet	Idaho 2008	1	.	11	ID
**1937**	Rambouillet	Idaho 2008	1	.	11	II
**1938**	Rambouillet	Idaho 2008	1	.	11	ID
**1939**	Rambouillet	Idaho 2008	4	.	11	II
**1940**	Rambouillet	Idaho 2008	1	.	11	II
**1941**	Rambouillet	Idaho 2008	1	.	11	ID
**1942**	Rambouillet	Idaho 2008	1	.	11	ID
**1943**	Rambouillet	Idaho 2008	1	.	11	II
**1944**	Rambouillet	Idaho 2008	4	.	11	ID
**1945**	Rambouillet	Idaho 2008	1	.	11	DD
**1946**	Rambouillet	Idaho 2008	4	.	11	DD
**1947**	Rambouillet	Idaho 2008	1	.	11	ID
**1948**	Rambouillet	Idaho 2008	4	.	11	ID
**1949**	Rambouillet	Idaho 2008	4	.	11	II
**1950**	Rambouillet	Idaho 2008	4	.	11	II
**1951**	Rambouillet	Idaho 2008	4	.	11	II
**1952**	Rambouillet	Idaho 2008	1	.	11	DD
**1953**	Rambouillet	Idaho 2008	1	.	11	II
**1954**	Rambouillet	Idaho 2008	4	.	11	ID
**1955**	Rambouillet	Idaho 2008	4	.	11	II
**1956**	Rambouillet	Idaho 2008	4	.	11	II
**1957**	Rambouillet	Idaho 2008	2	.	11	II
**1958**	Rambouillet	Idaho 2008	2	.	11	II
**1959**	Rambouillet	Idaho 2008	2	.	11	II
**1960**	Rambouillet	Idaho 2008	2	.	11	II
**1961**	Rambouillet	Idaho 2008	2	.	11	ID
**1962**	Rambouillet	Idaho 2008	2	.	11	DD
**1963**	Rambouillet	Idaho 2008	2	.	11	II
**1964**	Rambouillet	Idaho 2008	2	.	11	ID
**1965**	Rambouillet	Idaho 2008	2	.	11	II
**1966**	Rambouillet	Idaho 2008	2	.	11	ID
**1967**	Rambouillet	Idaho 2008	2	.	11	II
**1968**	Rambouillet	Idaho 2008	2	.	11	II
**1969**	Rambouillet	Idaho 2008	2	.	11	ID

**1970**	Rambouillet	Idaho 2008	2	.	11	II
**1971**	Rambouillet	Idaho 2008	2	.	11	ID
**1972**	Rambouillet	Idaho 2008	2	.	11	II
**1973**	Rambouillet	Idaho 2008	2	.	11	II
**1974**	Rambouillet	Idaho 2008	2	.	11	II
**1975**	Rambouillet	Idaho 2008	2	.	11	II
**1976**	Rambouillet	Idaho 2008	2	.	11	ID
**1977**	Rambouillet	Idaho 2008	2	.	11	ID
**1978**	Rambouillet	Idaho 2008	2	.	11	ID
**1979**	Rambouillet	Idaho 2008	2	.	11	ID
**1980**	Rambouillet	Idaho 2008	2	.	11	II
**1981**	Rambouillet	Idaho 2008	2	.	11	II
**1982**	Rambouillet	Idaho 2008	2	.	11	II
**1983**	Rambouillet	Idaho 2008	2	.	11	II
**1984**	Rambouillet	Idaho 2008	2	.	11	II
**1985**	Rambouillet	Idaho 2008	2	.	11	ID
**1986**	Rambouillet	Idaho 2008	2	.	11	II
**1987**	Rambouillet	Idaho 2008	2	.	11	ID
**1988**	Rambouillet	Idaho 2008	2	.	11	II
**1989**	Rambouillet	Idaho 2008	2	.	11	ID
**1990**	Rambouillet	Idaho 2008	3	.	11	II
**1991**	Rambouillet	Idaho 2008	2	.	11	II
**1992**	Rambouillet	Idaho 2008	2	.	11	ID
**1993**	Rambouillet	Idaho 2008	2	.	11	.
**1994**	Rambouillet	Idaho 2008	2	.	11	.
**1995**	Rambouillet	Idaho 2008	2	.	11	ID
**1996**	Rambouillet	Idaho 2008	2	.	11	.
**1997**	Rambouillet	Idaho 2008	2	.	11	ID
**1998**	Rambouillet	Idaho 2008	2	.	11	II
**1999**	Rambouillet	Idaho 2008	2	.	11	ID
**2000**	Rambouillet	Idaho 2008	2	.	11	II
**2001**	Rambouillet	Idaho 2008	2	.	11	II
**2002**	Rambouillet	Idaho 2008	2	.	11	II
**2003**	Rambouillet	Idaho 2008	2	.	11	II
**2004**	Rambouillet	Idaho 2008	2	.	11	.
**2005**	Rambouillet	Idaho 2008	2	.	11	II
**2006**	Rambouillet	Idaho 2008	2	.	11	II
**2007**	Rambouillet	Idaho 2008	2	.	11	II
**2008**	Rambouillet	Idaho 2008	2	.	11	II
**2009**	Rambouillet	Idaho 2008	2	.	11	II
**2010**	Rambouillet	Idaho 2008	2	.	11	II
**2011**	Rambouillet	Idaho 2008	2	.	11	ID
**2012**	Rambouillet	Idaho 2008	2	.	11	ID

**2013**	Rambouillet	Idaho 2008	2	.	11	.
**2014**	Rambouillet	Idaho 2008	2	.	11	DD
**2015**	Rambouillet	Idaho 2008	2	.	11	ID
**2016**	Rambouillet	Idaho 2008	2	.	11	II
**2017**	Rambouillet	Idaho 2008	2	.	11	II
**2018**	Rambouillet	Idaho 2008	2	.	11	II
**2019**	Rambouillet	Idaho 2008	2	.	11	II
**2020**	Rambouillet	Idaho 2008	2	.	11	II
**2021**	Rambouillet	Idaho 2008	2	.	11	DD
**2022**	Rambouillet	Idaho 2008	2	.	11	.
**2023**	Rambouillet	Idaho 2008	2	.	11	II
**2024**	Rambouillet	Idaho 2008	2	.	11	II
**2025**	Rambouillet	Idaho 2008	2	.	11	II
**2026**	Rambouillet	Idaho 2008	3	.	11	II
**2027**	Rambouillet	Idaho 2008	3	.	11	II
**2028**	Rambouillet	Idaho 2008	3	.	11	II
**2029**	Rambouillet	Idaho 2008	3	.	11	ID
**2030**	Rambouillet	Idaho 2008	3	.	11	II
**2031**	Rambouillet	Idaho 2008	3	.	11	II
**2032**	Rambouillet	Idaho 2008	3	.	11	ID
**2033**	Rambouillet	Idaho 2008	3	.	11	ID
**2034**	Rambouillet	Idaho 2008	3	.	11	ID
**2035**	Rambouillet	Idaho 2008	3	.	11	ID
**2036**	Rambouillet	Idaho 2008	3	.	11	ID
**2037**	Rambouillet	Idaho 2008	3	.	11	ID
**2038**	Rambouillet	Idaho 2008	3	.	11	ID
**2039**	Rambouillet	Idaho 2008	3	.	11	.
**2040**	Rambouillet	Idaho 2008	3	.	11	ID
**2041**	Rambouillet	Idaho 2008	3	.	11	ID
**2042**	Rambouillet	Idaho 2008	3	.	11	ID
**2043**	Rambouillet	Idaho 2008	3	.	11	II
**2044**	Rambouillet	Idaho 2008	3	.	11	II
**2045**	Rambouillet	Idaho 2008	3	.	11	ID
**2046**	Rambouillet	Idaho 2008	3	.	11	II
**2047**	Rambouillet	Idaho 2008	3	.	11	ID
**2048**	Rambouillet	Idaho 2008	3	.	11	II
**2049**	Rambouillet	Idaho 2008	3	.	11	II
**2050**	Rambouillet	Idaho 2008	3	.	11	ID
**2051**	Rambouillet	Idaho 2008	3	.	11	II
**2052**	Rambouillet	Idaho 2008	3	.	11	ID
**2053**	Rambouillet	Idaho 2008	3	.	11	ID
**2054**	Rambouillet	Idaho 2008	3	.	11	ID
**2055**	Rambouillet	Idaho 2008	3	.	11	II

**2056**	Rambouillet	Idaho 2008	3	.	11	II
**2057**	Rambouillet	Idaho 2008	3	.	11	II
**2058**	Rambouillet	Idaho 2008	3	.	11	II
**2059**	Rambouillet	Idaho 2008	3	.	11	DD
**2060**	Rambouillet	Idaho 2008	3	.	11	DD
**2061**	Rambouillet	Idaho 2008	3	.	11	DD
**2062**	Rambouillet	Idaho 2008	3	.	11	II
**2063**	Rambouillet	Idaho 2008	3	.	11	ID
**2064**	Rambouillet	Idaho 2008	3	.	11	DD
**2065**	Rambouillet	Idaho 2008	3	.	11	ID
**2066**	Rambouillet	Idaho 2008	3	.	11	ID
**2067**	Rambouillet	Idaho 2008	3	.	11	DD
**2068**	Rambouillet	Idaho 2008	3	.	14	ID
**2069**	Rambouillet	Idaho 2008	3	.	14	ID
**2070**	Rambouillet	Idaho 2008	1	.	14	II
**2071**	Rambouillet	Idaho 2008	4	.	110	II
**2072**	Rambouillet	Idaho 2008	4	.	110	ID
**2073**	Rambouillet	Idaho 2008	2	1.01	11	II
**2074**	Rambouillet	Idaho 2008	4	1.05	11	ID
**2075**	Rambouillet	Idaho 2008	2	1.05	11	ID
**2076**	Rambouillet	Idaho 2008	1	1.17	11	ID
**2077**	Rambouillet	Idaho 2008	1	1.25	11	II
**2078**	Rambouillet	Idaho 2008	1	1.65	11	II
**2079**	Rambouillet	Idaho 2008	4	10.2	11	II
**2080**	Rambouillet	Idaho 2008	2	10.3	11	II
**2081**	Rambouillet	Idaho 2008	2	10.4	13	II
**2082**	Rambouillet	Idaho 2008	2	10.9	11	II
**2083**	Rambouillet	Idaho 2008	4	102	11	ID
**2084**	Rambouillet	Idaho 2008	4	1030	11	II
**2085**	Rambouillet	Idaho 2008	2	11.8	11	ID
**2086**	Rambouillet	Idaho 2008	2	110	11	ID
**2087**	Rambouillet	Idaho 2008	1	12.2	11	II
**2088**	Rambouillet	Idaho 2008	4	12.8	11	ID
**2089**	Rambouillet	Idaho 2008	2	121	11	II
**2090**	Rambouillet	Idaho 2008	2	1210	11	II
**2091**	Rambouillet	Idaho 2008	5	123	11	II
**2092**	Rambouillet	Idaho 2008	2	13	11	II
**2093**	Rambouillet	Idaho 2008	3	13.9	11	ID
**2094**	Rambouillet	Idaho 2008	2	133	11	II
**2095**	Rambouillet	Idaho 2008	2	134	13	.
**2096**	Rambouillet	Idaho 2008	3	14	11	ID
**2097**	Rambouillet	Idaho 2008	4	14.2	11	ID
**2098**	Rambouillet	Idaho 2008	4	14.3	110	ID

**2099**	Rambouillet	Idaho 2008	4	147	13	ID
**2100**	Rambouillet	Idaho 2008	4	1490	11	ID
**2101**	Rambouillet	Idaho 2008	2	15	11	II
**2102**	Rambouillet	Idaho 2008	5	15	11	II
**2103**	Rambouillet	Idaho 2008	4	15.2	11	ID
**2104**	Rambouillet	Idaho 2008	3	15.9	11	ID
**2105**	Rambouillet	Idaho 2008	4	1550	11	.
**2106**	Rambouillet	Idaho 2008	4	16.4	11	ID
**2107**	Rambouillet	Idaho 2008	4	161	11	ID
**2108**	Rambouillet	Idaho 2008	2	161	11	II
**2109**	Rambouillet	Idaho 2008	4	17.3	11	ID
**2110**	Rambouillet	Idaho 2008	4	17.7	11	II
**2111**	Rambouillet	Idaho 2008	4	1760	11	.
**2112**	Rambouillet	Idaho 2008	3	178	11	.
**2113**	Rambouillet	Idaho 2008	2	179	11	ID
**2114**	Rambouillet	Idaho 2008	5	18	11	DD
**2115**	Rambouillet	Idaho 2008	3	18.4	11	II
**2116**	Rambouillet	Idaho 2008	3	19.4	11	II
**2117**	Rambouillet	Idaho 2008	5	190	11	DD
**2118**	Rambouillet	Idaho 2008	4	19700	11	ID
**2119**	Rambouillet	Idaho 2008	4	2.26	11	II
**2120**	Rambouillet	Idaho 2008	4	2.27	17	ID
**2121**	Rambouillet	Idaho 2008	3	2.86	11	II
**2122**	Rambouillet	Idaho 2008	4	2.95	11	II
**2123**	Rambouillet	Idaho 2008	4	2.97	11	ID
**2124**	Rambouillet	Idaho 2008	3	20.1	11	ID
**2125**	Rambouillet	Idaho 2008	2	20.1	11	ID
**2126**	Rambouillet	Idaho 2008	4	20.6	11	DD
**2127**	Rambouillet	Idaho 2008	1	20.7	11	.
**2128**	Rambouillet	Idaho 2008	3	209	11	II
**2129**	Rambouillet	Idaho 2008	1	2100	11	ID
**2130**	Rambouillet	Idaho 2008	4	216	11	.
**2131**	Rambouillet	Idaho 2008	4	223	11	ID
**2132**	Rambouillet	Idaho 2008	3	229	11	.
**2133**	Rambouillet	Idaho 2008	4	23.4	11	ID
**2134**	Rambouillet	Idaho 2008	3	248	13	II
**2135**	Rambouillet	Idaho 2008	4	25.1	11	.
**2136**	Rambouillet	Idaho 2008	4	2500	11	ID
**2137**	Rambouillet	Idaho 2008	4	251	110	II
**2138**	Rambouillet	Idaho 2008	4	2510	11	II
**2139**	Rambouillet	Idaho 2008	4	255	11	II
**2140**	Rambouillet	Idaho 2008	2	26.7	11	ID
**2141**	Rambouillet	Idaho 2008	4	27.3	11	ID

**2142**	Rambouillet	Idaho 2008	4	276	11	.
**2143**	Rambouillet	Idaho 2008	2	28.9	11	ID
**2144**	Rambouillet	Idaho 2008	2	29.9	11	II
**2145**	Rambouillet	Idaho 2008	4	297	11	II
**2146**	Rambouillet	Idaho 2008	2	3.14	11	II
**2147**	Rambouillet	Idaho 2008	2	3.24	11	ID
**2148**	Rambouillet	Idaho 2008	3	3.62	11	ID
**2149**	Rambouillet	Idaho 2008	3	31.7	11	ID
**2150**	Rambouillet	Idaho 2008	3	32.5	11	II
**2151**	Rambouillet	Idaho 2008	4	32.5	11	II
**2152**	Rambouillet	Idaho 2008	4	329	11	II
**2153**	Rambouillet	Idaho 2008	4	33.2	11	II
**2154**	Rambouillet	Idaho 2008	4	33.7	11	ID
**2155**	Rambouillet	Idaho 2008	4	332	11	ID
**2156**	Rambouillet	Idaho 2008	3	34.5	11	II
**2157**	Rambouillet	Idaho 2008	2	34.7	11	II
**2158**	Rambouillet	Idaho 2008	1	34.8	11	ID
**2159**	Rambouillet	Idaho 2008	3	3420	11	II
**2160**	Rambouillet	Idaho 2008	3	35.1	11	ID
**2161**	Rambouillet	Idaho 2008	4	351	11	ID
**2162**	Rambouillet	Idaho 2008	2	36.8	11	II
**2163**	Rambouillet	Idaho 2008	2	362	11	ID
**2164**	Rambouillet	Idaho 2008	2	3690	11	ID
**2165**	Rambouillet	Idaho 2008	4	37.5	11	ID
**2166**	Rambouillet	Idaho 2008	4	37.8	11	II
**2167**	Rambouillet	Idaho 2008	4	37.8	11	ID
**2168**	Rambouillet	Idaho 2008	1	372	13	II
**2169**	Rambouillet	Idaho 2008	3	38.6	11	II
**2170**	Rambouillet	Idaho 2008	2	38.9	11	II
**2171**	Rambouillet	Idaho 2008	4	39.8	13	II
**2172**	Rambouillet	Idaho 2008	2	4.28	11	II
**2173**	Rambouillet	Idaho 2008	4	4.29	11	ID
**2174**	Rambouillet	Idaho 2008	3	4.3	110	II
**2175**	Rambouillet	Idaho 2008	5	4.45	11	ID
**2176**	Rambouillet	Idaho 2008	3	4.91	11	II
**2177**	Rambouillet	Idaho 2008	4	40.4	11	DD
**2178**	Rambouillet	Idaho 2008	4	400	11	ID
**2179**	Rambouillet	Idaho 2008	2	405	11	ID
**2180**	Rambouillet	Idaho 2008	4	405	11	ID
**2181**	Rambouillet	Idaho 2008	3	413	11	ID
**2182**	Rambouillet	Idaho 2008	4	44.2	11	II
**2183**	Rambouillet	Idaho 2008	3	44.4	11	ID
**2184**	Rambouillet	Idaho 2008	5	4440	11	II

**2185**	Rambouillet	Idaho 2008	3	449	11	II
**2186**	Rambouillet	Idaho 2008	4	45.5	13	II
**2187**	Rambouillet	Idaho 2008	4	48.4	11	DD
**2188**	Rambouillet	Idaho 2008	1	48.4	11	ID
**2189**	Rambouillet	Idaho 2008	2	49.7	11	II
**2190**	Rambouillet	Idaho 2008	4	5.79	11	II
**2191**	Rambouillet	Idaho 2008	5	5.87	11	DD
**2192**	Rambouillet	Idaho 2008	2	50	11	II
**2193**	Rambouillet	Idaho 2008	4	500	11	II
**2194**	Rambouillet	Idaho 2008	3	504	11	DD
**2195**	Rambouillet	Idaho 2008	3	52	11	ID
**2196**	Rambouillet	Idaho 2008	3	53.1	11	ID
**2197**	Rambouillet	Idaho 2008	4	54	11	.
**2198**	Rambouillet	Idaho 2008	2	54.7	11	.
**2199**	Rambouillet	Idaho 2008	2	55.2	11	II
**2200**	Rambouillet	Idaho 2008	4	5620	11	.
**2201**	Rambouillet	Idaho 2008	4	589	11	II
**2202**	Rambouillet	Idaho 2008	2	6.04	11	II
**2203**	Rambouillet	Idaho 2008	4	6.36	11	ID
**2204**	Rambouillet	Idaho 2008	4	6.64	11	ID
**2205**	Rambouillet	Idaho 2008	3	6.95	11	II
**2206**	Rambouillet	Idaho 2008	4	60.3	11	ID
**2207**	Rambouillet	Idaho 2008	2	60.4	11	II
**2208**	Rambouillet	Idaho 2008	1	60.6	11	II
**2209**	Rambouillet	Idaho 2008	4	60.8	11	.
**2210**	Rambouillet	Idaho 2008	3	61.2	11	ID
**2211**	Rambouillet	Idaho 2008	4	610	11	II
**2212**	Rambouillet	Idaho 2008	5	64	11	ID
**2213**	Rambouillet	Idaho 2008	3	64.2	11	ID
**2214**	Rambouillet	Idaho 2008	4	64.4	13	II
**2215**	Rambouillet	Idaho 2008	4	655	11	DD
**2216**	Rambouillet	Idaho 2008	4	66	11	II
**2217**	Rambouillet	Idaho 2008	2	66.8	11	II
**2218**	Rambouillet	Idaho 2008	3	67	11	ID
**2219**	Rambouillet	Idaho 2008	2	7.01	110	II
**2220**	Rambouillet	Idaho 2008	4	70.1	11	ID
**2221**	Rambouillet	Idaho 2008	4	70.8	11	DD
**2222**	Rambouillet	Idaho 2008	4	744	110	ID
**2223**	Rambouillet	Idaho 2008	5	79	11	.
**2224**	Rambouillet	Idaho 2008	4	792	11	ID
**2225**	Rambouillet	Idaho 2008	3	8.14	11	II
**2226**	Rambouillet	Idaho 2008	2	8.56	11	.
**2227**	Rambouillet	Idaho 2008	2	85.9	11	II

**2228**	Rambouillet	Idaho 2008	3	87.3	11	ID
**2229**	Rambouillet	Idaho 2008	4	9.86	11	II
**2230**	Rambouillet	Idaho 2008	5	90.9	11	ID
**2231**	Rambouillet	Idaho 2008	4	91.1	11	II
**2232**	Rambouillet	Idaho 2008	5	92	11	II
**2233**	Rambouillet	Idaho 2008	5	9550	13	ID
**2234**	Rambouillet	Idaho 2008	4	967	11	II
**2235**	Rambouillet	Idaho 2008	2	98.2	11	II
**2236**	Rambouillet	Idaho 2008	4	993	11	DD

II: Insertion homozygote
DD: Deletion homozygote
ID: Insertion-Deletion heterozygote
.: Data missing
